# Genetic variants for smoking behaviour and risk of skin cancer

**DOI:** 10.1038/s41598-023-44144-0

**Published:** 2023-10-06

**Authors:** Jean Claude Dusingize, Matthew H. Law, Mathias Seviiri, Catherine M. Olsen, Nirmala Pandeya, Maria Teresa Landi, Mark M. Iles, Rachel E. Neale, Jue-Sheng Ong, Stuart MacGregor, David C. Whiteman

**Affiliations:** 1 Departments of Population Health and Computational Biology, QIMR Berghofer Medical Research Institute, Brisbane, QLD Australia; 2 School of Biomedical Sciences, Faculty of Health, Queensland University of Technology, Brisbane, QLD Australia; 3 Faculty of Medicine, The University of Queensland, Brisbane, QLD Australia; 4 Division of Cancer Epidemiology and Genetics, National Cancer Institute, National Institutes of Health, Bethesda, MD USA; 5 Leeds Institute for Data Analytics, University of Leeds, Leeds, UK

**Keywords:** Genetics, Cancer, Cancer genetics, Cancer prevention, Skin cancer

## Abstract

Observational studies have suggested that smoking may increase the risk of cutaneous squamous cell carcinoma (cSCC) while decreasing the risks of basal cell carcinoma (BCC), and melanoma. However, it remains possible that confounding by other factors may explain these associations. The aim of this investigation was to use Mendelian randomization (MR) to test whether smoking is associated with skin cancer, independently of other factors. Two-sample MR analyses were conducted to determine the causal effect of smoking measures on skin cancer risk using genome-wide association study (GWAS) summary statistics. We used the inverse-variance-weighted estimator to derive separate risk estimates across genetic instruments for all smoking measures. A genetic predisposition to smoking initiation was associated with lower risks of all skin cancer types, although none of the effect estimates reached statistical significance (OR 95% CI BCC 0.91, 0.82–1.01; cSCC 0.82, 0.66–1.01; melanoma 0.91, 0.82–1.01). Results for other measures were similar to smoking initiation with the exception of smoking intensity which was associated with a significantly reduced risk of melanoma (OR 0.67, 95% CI 0.51–0.89). Our findings support the findings of observational studies linking smoking to lower risks of melanoma and BCC. However, we found no evidence that smoking is associated with an elevated risk of cSCC; indeed, our results are most consistent with a decreased risk, similar to BCC and melanoma.

## Introduction

Tobacco smoke contains carcinogenic substances that damage the DNA of cells and cause mutations^1^. These mutations disrupt normal cellular processes and lead to uncontrolled growth. Over time, these genetic alterations can result in the formation of cancerous tumours. The International Agency for Research on Cancer (IARC) has linked tobacco smoking to numerous cancers in humans^2–4^. However, the role of smoking in the development of skin cancer is not well understood despite extensive investigations. The association between smoking and the risk of developing cutaneous squamous cell carcinoma (cSCC) and basal cell carcinoma (BCC) has been previously evaluated by two meta-analyses comprising cohort and case–control studies; both reported inconsistent findings. The first meta-analysis reported that “ever” smokers were at slightly increased risk of BCC (OR 1.02, 95% CI 1.00–1.04) and cSCC (OR 1.08, 95% CI 1.01–1.15) relative to never smokers^5^. The second meta-analysis reported a significant 50% increase in the risk of cSCC to ever smokers, but no association was seen with BCC (OR, 0.95, 95% CI 0.82–1.09)^6^. More recently, the United Kingdom Million Women Study found that in individuals who currently smoke compared to those who have never smoked, there was an elevated incidence of SCC (RR 1.22, 95% CI 1.15–1.31), while the incidence of BCC decreased (RR 0.80, 95% CI 0.78–0.82)^7^. However, this study was conducted only among women and so may not be generalizable to men. Data from the QSkin study, a prospective cohort in Australia, purpose-designed to investigate skin cancer outcomes, also found that current smokers had significantly higher risks of cSCC but lower risks of BCC^8^.

For melanoma, most studies have reported moderate inverse associations with smoking. For example, a meta-analysis restricted to prospective cohort studies found that men who were ever smokers had a significant 30% decrease in the risk of melanoma compared to men who never smoked, while the association was null for women (relative risk (RR) 0.96; 95% CI 0.83–1.10)^5^. A second meta-analysis including both cohort and case–control studies also reported a significant 30% decrease in the risk of melanoma among current smokers compared with never smokers, but that meta-analysis did not report sex-specific estimates^9^. Findings from the QSkin cohort showed that, compared with never smokers, former smokers had a significantly lower risk of melanoma, but no significant association was found for current smokers (RR 1.01; 95% CI 0.64–1.61)^10^.

Given that much of the current evidence stems from observational studies, the causal nature of these findings cannot be determined as there are complex webs of confounding that exist between smoking, other socio-demographic and behavioral factors, ultraviolet radiation exposure, and skin cancer that cannot be adequately accounted for through statistical adjustment. Randomized controlled trials (RCTs) are, in principle, the gold standard design for determining the effect of a specific exposure on an outcome. Moreover, RCT designs are not ethically feasible for smoking exposures. Given the inconsistent findings from the observational studies, and the unfeasibility of an RCT, alternative approaches are needed to bring clarity to the previously reported associations between smoking and skin cancer.

Mendelian randomisation (MR) is an analytic approach that utilizes genetic data to overcome confounding in observational studies^11^. MR uses genetic variants such as single nucleotide polymorphisms (SNPs) associated with a trait of interest as instrumental variables to assess associations between an exposure and a disease outcome^11^. Because genetic factors randomly segregate when passed from parents to offspring, MR studies can be regarded as naturally occurring RCTs in which genetic alleles are randomly assigned to children from parental genotypes^12^. The risk factors predicted by genetic variants are not confounded by other exposures (provided certain assumptions hold), and reverse causality is avoided as an individual’s genotype is assigned at conception and cannot be modified by disease development. Numerous twin and family studies^13,14^ have demonstrated that about 50% of the phenotypic variance in nicotine dependence and smoking behaviors are attributable to genetic factors. Genome-wide association studies (GWAS) have identified several single-nucleotide polymorphisms (SNPs) associated with smoking behaviors^15,16^. These SNPs can be used as instruments to infer causality in an MR framework.

A recent MR study within the UK biobank including 4869 melanoma cases reported an inverse but non-significant association between smoking initiation and risk of melanoma (OR 0.92, 95% CI 0.80–1.06)^17^. However, there was no assessment of the effects of other measures of smoking behavior such as the number of cigarettes smoked per day, smoking duration and intensity, or smoking cessation. The recent release of the melanoma GWAS meta-analysis comprising 36,760 melanoma cases^18^ offers an avenue for this important research question to be revisited. In the light of the continuing uncertainty about the role of smoking in skin cancer development, and given these new sources of high-quality genetic data, we sought to conduct a new investigation to quantify the role of smoking using MR methods.

## Results

### Validation of genetic instruments for smoking measures

The genetic variants that we used for these analyses were robust instruments and independently predicted the likelihood of being a smoker in the QSkin cohort; the relative risk of being a smoker was twofold higher among people in the top decile of the smoking initiation PRS compared to those in the lowest decile (OR 95% CI 1.98, 1.72–2.28) (Supplementary Fig. S1). The associations were less strong for lifetime smoking index (LSI) (OR 1.51, 95% CI 1.31–1.73) and smoking intensity (OR 1.18, 95% CI 1.03–1.36), possibly due to the reduced statistical power as the analyses involving those two parameters were restricted to ever-smokers (n = 7009) (Supplementary Fig. S1).

### Mendelian randomization estimates for smoking initiation

**Figure 1:**
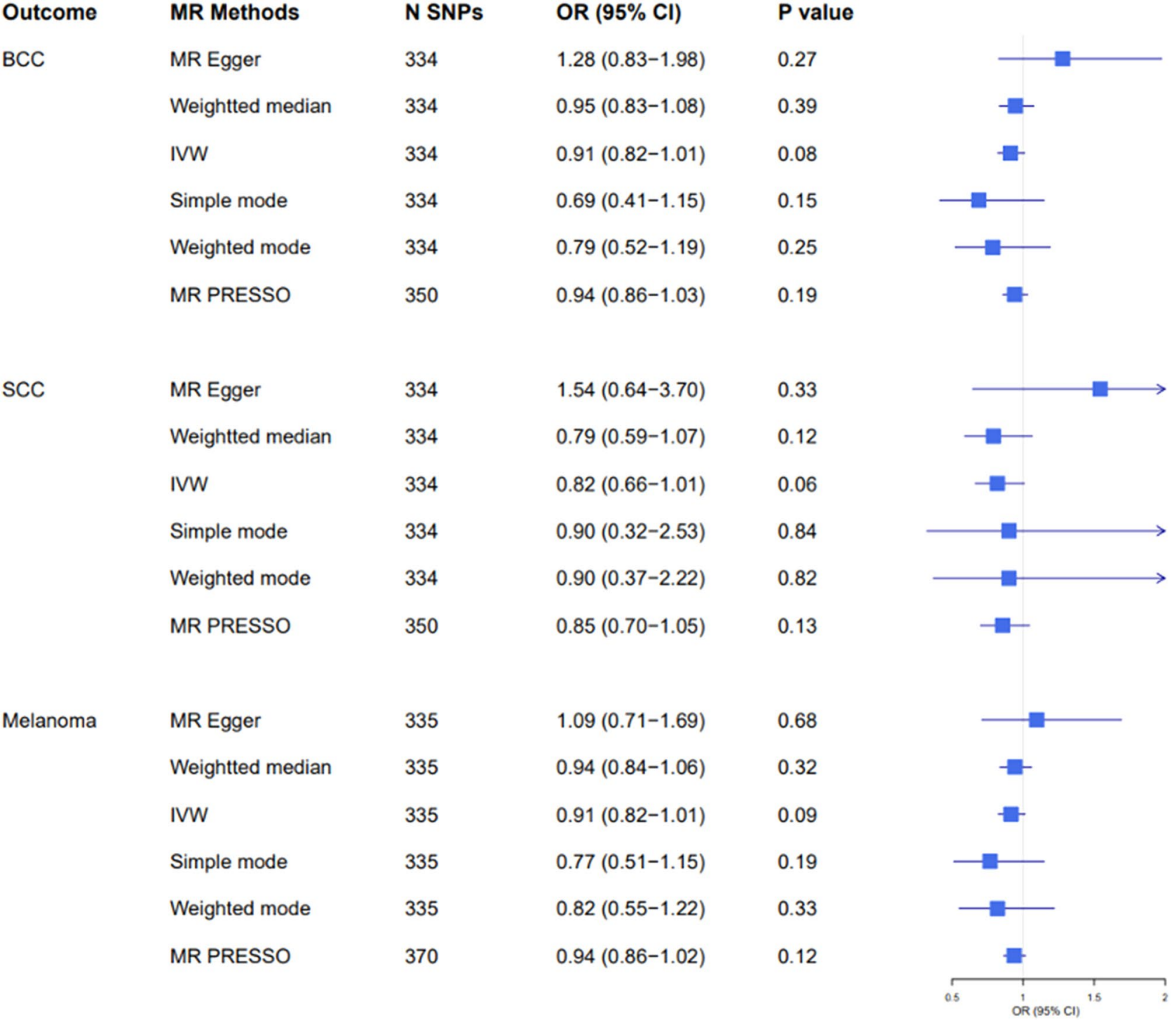
Two-sample Mendelian randomisation analyses for the associations between genetic predisposition to smoking initiation and risk of developing basal cell carcinoma (BCC), squamous cell carcinoma (SCC), and melanoma. *OR* odds ratio, *CI* confidence interval. *N SNPs* number of single nucleotide polymorphisms.

A total of 378 genetic variants obtained from a previously published study were selected as genetic instruments for ever having smoked regularly. We excluded palindromic variants and variants that could not be obtained from the current skin cancer GWAS datasets, leaving 334 variants for MR analyses for BCC and cSCC and 355 for the analysis of melanoma. The IVW model showed that while none of the estimates reached statistical significance, genetic predisposition to smoking initiation was associated with lower risk of developing skin cancer OR, 95% CI 0.91, 0.82–1.01, *p* = 0.08 for BCC; 0.82, 0.66–1.01, *p* = 0.06 for cSCC and 0.91, 0.82–1.01, *p* = 0.09 for melanoma (Fig. 1 and Supplementary Fig. S2). The direction of the effect estimates was consistent across all MR methods, with the exception of MR-Egger, for which, a modest but non-statistically significant positive effect was found. Although the MR-Egger estimate was in the opposite direction, the confidence intervals were wide. We contend that this single finding does not contradict evidence obtained from IVW method although it provides no additional evidence of causal effect^19^. We found no evidence of heterogeneity in the effect sizes of the genetic variants employed and MR-Egger intercepts were virtually null, suggesting that there was no directional pleiotropy (Supplementary Table S1). MR PRESSO identified two outlier variants for BCC, one variant for cSCC and three variants for melanoma; the risk estimates remained unchanged after removing those variants (Fig. 1).

### Mendelian randomization estimates for smoking intensity

**Figure 2:**
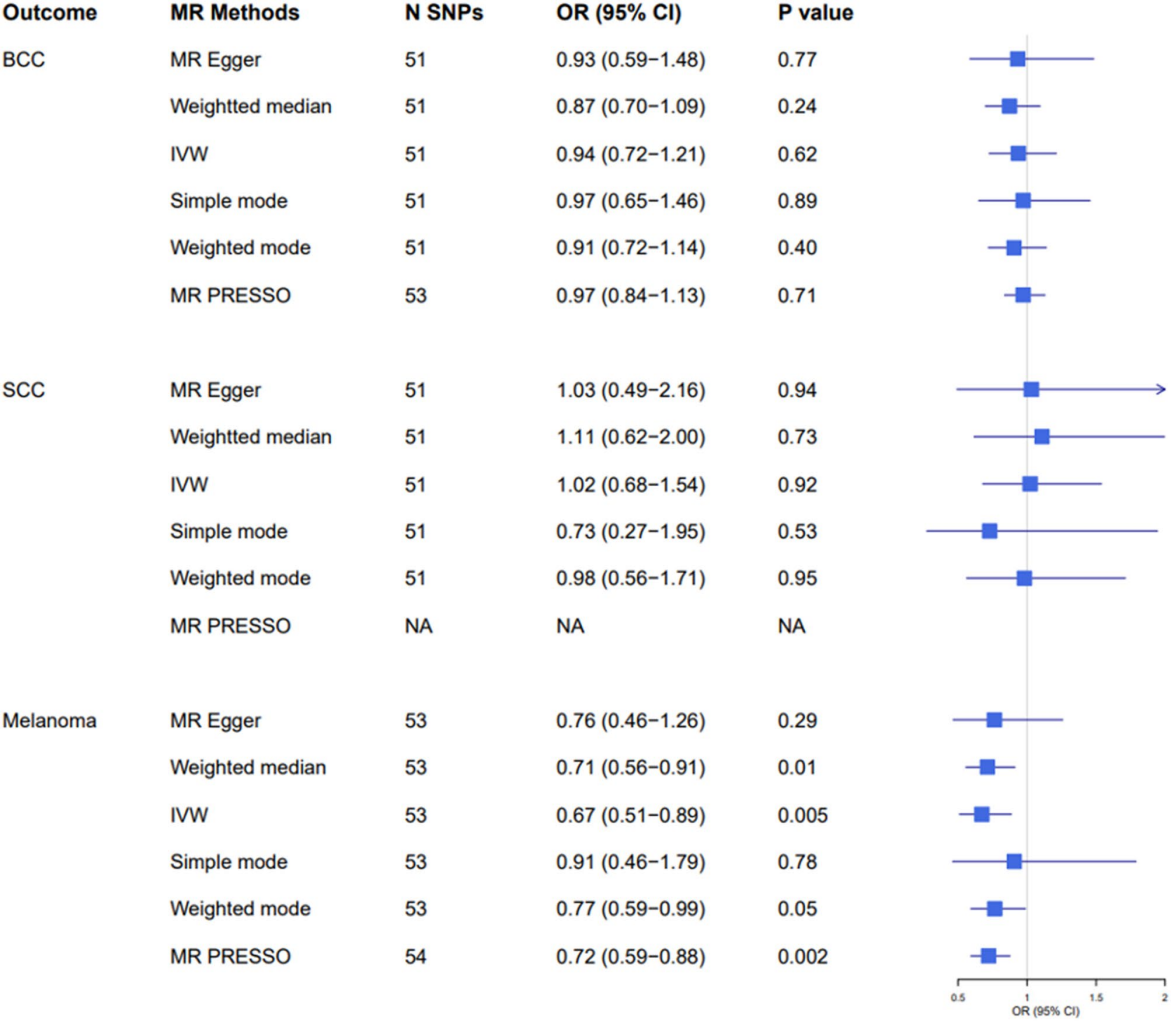
Two-sample Mendelian randomisation analyses for the associations between smoking intensity and risk of developing basal cell carcinoma (BCC), squamous cell carcinoma (SCC), and melanoma. *OR* odds ratio, *CI* confidence interval. *N SNPs* number of single nucleotide polymorphisms; *NA* the MR-PRESSO method did not detect any outlier variant for SCC.

The genetic instruments for the number of cigarettes smoked per day included 55 variants. After excluding palindromic variants and variants that could not be identified from the skin cancer GWASs, 51 variants remained for the MR analyses for BCC and cSCC and 53 for the MR analysis for melanoma. Using the IVW model, we estimated the overall OR of developing BCC, cSCC, and melanoma per 1 SD increase in the number of cigarettes smoked per day. Based on combined estimates derived from all variants, we found an inverse but non-significant association between genetically predicted smoking intensity and risk of developing BCC (OR 0.94, 95% CI 0.72–1.21); a slightly increased but non-significant association was found for cSCC (OR 1.02, 95% CI 0.68–1.54). We found however, strong evidence that genetically predicted smoking intensity was associated with significantly reduced risk of melanoma (OR 0.67, 95% CI 0.51–0.89, *p* = 0.005) (Fig. 2 and Supplementary Fig. S3). The direction of effect estimates for melanoma were consistent across all MR sensitivity analyses and MR-Egger showed little evidence of pleiotropy. The MR-PRESSO method detected two outlier variants for BCC and one variant for melanoma, but the main results did not change after correcting for those variants. (Fig. 2). The MR-PRESSO method did not detect any outlier variant for cSCC.

### Mendelian randomization estimates for lifetime amount of smoking

**Figure 3:**
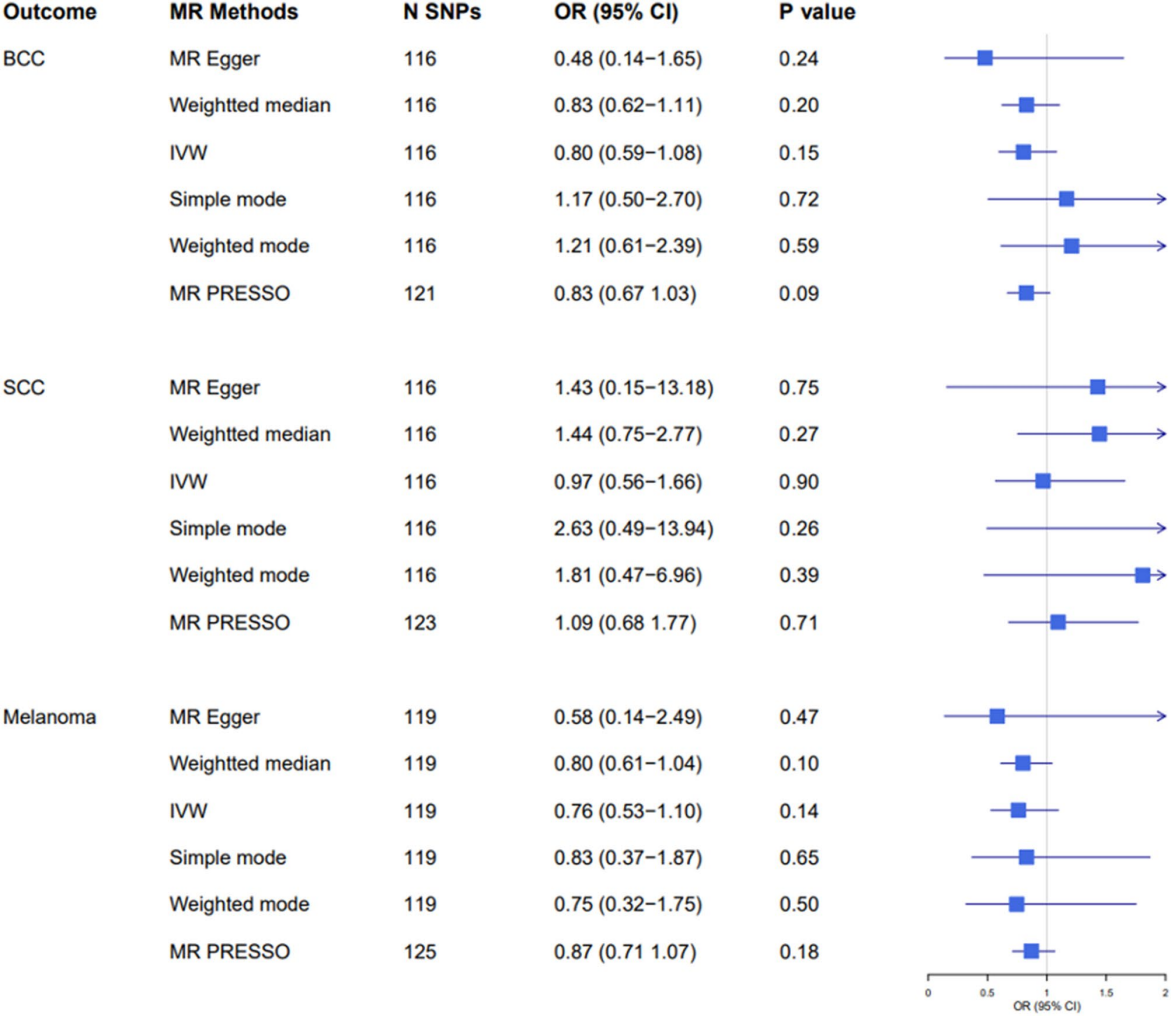
Two-sample Mendelian randomisation analyses for the associations between lifetime smoking index and risk of developing basal cell carcinoma (BCC), squamous cell carcinoma (SCC), and melanoma. *OR* odds ratio, *CI* confidence interval. *N SNPs*: number of single nucleotide polymorphisms.

From the LSI GWAS, we selected 126 independent variants as genetic instruments. We excluded 10 variants for MR analyses for BCC and cSCC and seven variants for MR analysis for melanoma for being either palindromic or absent in the existing skin cancer GWAS datasets. In the IVW model, we found inverse, but non-statistically significant associations between genetically predicted LSI and risk of BCC and melanoma, (OR per 1 SD increment in LSI, 95% CI 0.80, 0.59–1.08 for BCC and 0.76, 0.53–1.10 for melanoma) (Fig. 3 and Supplementary Fig. S4). The effect estimate for cSCC was also inverse but closer to null (OR 0.97, 95% CI 0.56–1.66). MR-Egger analyses showed no evidence of directional pleiotropy and outlier-corrected estimates from MR-PRESSO also indicated that our findings were not influenced by outlier variants.

## Discussion

In this MR study, we investigated the role of smoking in the development of skin cancer using summary data from the largest GWAS meta-analyses to date. Collectively, we found weakly inverse associations between genetic predisposition to smoking and risk of developing all three common types of skin cancer. Our findings support earlier observational studies reporting that smoking is associated with a lower risk of melanoma and BCC. However, despite numerous pooled analyses from observational studies that reported increased risk of cSCC associated with current compared to never smokers, our genetic analyses found no evidence that smoking is associated with elevated risk of cSCC.

Smoking is a complex phenotype determined by environmental and genetic factors, and their interactions^20^. Numerous studies have documented that variation in genetic background is an important determinant of smoking-related behaviour. Previous studies employing the MR approach, including a systematic review and meta-analysis of MR studies^21^, have demonstrated that genetic liability to smoking initiation or lifetime smoking is associated with risk of developing several cancers such as lung, bladder, oesophagus, and head and neck^17,21^. Such findings lend further credibility to support the use of genetic instruments as unbiased measures for smoking. To our knowledge, no prior MR studies have investigated the relationship between smoking initiation and BCC or cSCC, and only one study (using UK Biobank data) has examined associations between genetic predisposition to regular smoking and melanoma risk. The reported estimates from that study were consistent with our findings (OR 0.92, 95% CI 0.80–1.06)^17^. We also used additional measures of smoking (smoking intensity and LSI) which have not been analysed previously to assess skin cancer risk.

The lower risks of melanoma and BCC documented in our study among smokers are consistent with previous prospective observational studies, including our own investigations in the QSkin cohort^8,10^. For example, a recent meta-analysis of cohort studies found that current smoking and heavy smoking were inversely associated with BCC and melanoma [pooled relative risk (RR) for BCC 0.85, 95% CI 0.75–0.96 and pooled RR for melanoma 0.72, 95% CI 0.64–0.82)]. However, it was unclear whether those relationships were causal since observational studies can suffer from bias and confounding that may be difficult to account for through typical multivariable regression analyses. The inverse associations observed in our genetic study for BCC and melanoma, albeit modest, cannot be explained by residual confounding.

The carcinogenic effect of tobacco smoking is well established, and the International Agency for Research on Cancer (IARC) has linked tobacco smoking to numerous cancers in humans^3^. However, tobacco smoking has also been consistently associated with reduced risks of some conditions such as Parkinson’s disease (PD)^22^ and ulcerative colitis^23^. The mechanisms that underlie the lower risks of those conditions among smokers remain unclear. It has been established that tobacco smoking releases many chemicals and by-products into the airways, many of which have biologic activity^1^. While most of the literature has focussed on the pharmacologic effects of nicotine on particular cells, there are many other factors in tobacco smoke that might influence tumorigenesis in pigment cells. Human melanocytes cultured with tobacco smoke extract showed higher levels of pigmentation than cells without tobacco smoke extract^24^. Animal studies have also documented nicotine’s affinity for melanin-containing tissues^25,26^. Thus, we can speculate that perhaps a selective effect of tobacco smoke on melanocyte activity may explain, at least in part, a possible role of smoking in inhibiting the development of melanoma. The extracts of tobacco may also inhibit the growth of BCC by downregulating the Notch Pathway, an important gene implicated in differentiation and growth of keratinocytes^27^.

Despite early pooled analyses from observational studies that support a causal role of smoking in the development of cSCC, we found no evidence that genetic predisposition to smoking is associated with elevated risk of cSCC. Indeed, on the contrary, we observed a weak inverse association. Most observational studies evaluating the role of smoking on cSCC, however, reported mixed findings for current versus former smokers and did not find significant dose–response relationships, which argue against causal effects of smoking on increased risk of cSCC. The apparently paradoxical findings of a negative association between smoking and BCC, and a null funding with SCC, may stem from a variety of factors. For example, it is thought that BCC and SCC originate from different types of skin cells. Smoking could impact epidermal cells in the basal layer differently from the squamous layer, thus contributing to the observed differential association. The lack of causal association between smoking and cSCC observed here has led us to propose that other non-causal explanations such as bias or confounding are more likely to drive the increased risk of cSCC among smokers reported in prior observational studies. It is possible, indeed likely, that factors that are linked both to smoking and SCC risk, such as education or occupation, might confound the association. Publication bias might also play a role since studies reporting a significant association between smoking and SCC are more likely to be published.

Our investigation presents a number of strengths. We performed two-sample MR using large GWAS datasets, offering higher statistical power than previously performed MR analyses. Any possible bias due to participant overlap was avoided as GWAS summary data for smoking and skin cancer were obtained from independent samples. Further, the genetic instruments for smoking measures were validated in an independent dataset, showing significant trends between increasing PRS for smoking initiation and LSI and the likelihood of being a regular smoker (Supplementary Fig. S1). The PRS for smoking intensity did not predict the likelihood of being a regular smoker in the QSkin cohort, however.

Our investigation was limited somewhat by statistical power. Specifically, we had low precision for some analyses, particularly for cSCC (Supplementary Table S2), owing to the low number of SCC cases and the small proportion of phenotypic variation in smoking-related traits. The genetic variants employed as instrumental variables explained only 2% of variance in smoking initiation, necessitating very large samples to detect small effects. Current GWAS studies have been crucial in identifying numerous genetic variants associated with smoking-related traits, but there is still a large proportion of genetic variance unaccounted for; this is referred to as “missing heritability.” Gene-environment interactions have been hypothesized as a potential source of missing heritability^28^, but it is also possible that genetic variants yet to be discovered may account for a considerable proportion. Sample size constraints also meant that we were unable to assess possible non-linear relationships between smoking and risk of skin cancer; such analyses are possible but would require much larger sample sizes. Our study may also have limited generalizability as our analyses included only individuals of European descent; however, the risk of skin cancer is low in other populations. Finally, although we examined nine hypotheses, we did not apply corrections for multiple testing. This decision was based on the fact that our analyses were driven by recent observational studies, making them hypothesis-driven and guided by a pre-established significance level. As such, the likelihood of obtaining false-positive results has been significantly reduced^29^.

In summary, using different MR methods, we have tested possible associations between smoking and risk of developing skin cancer. Collectively, we found that genetic predisposition to smoking does not increase the risk of developing any type of skin cancer. Instead, we observed weak inverse associations with smoking, particularly for BCC and melanoma. The lower risks of melanoma and BCC among smokers, which are consistent with pooled analyses from numerous observational studies, suggest that the associations between smoking and those cancers are likely causal. Our analyses do not support findings from observational studies suggesting that a positive association exists between smoking and cSCC risk, particularly among current smokers. We conclude that other non-causal explanations explain observed relationships between smoking and cSCC. While clearly smoking cannot ever be promoted as a means to reduce melanoma risk, there is merit to understanding the mechanisms through which smoking might mediate its putative inhibitory effect, with a view to possible chemoprevention. For example, investigating the differential gene expression patterns in skin tissues of smokers and non-smokers with these skin cancers could uncover molecular pathways that are affected by smoking and contribute to varying risks. Studies on epigenetic modifications, such as DNA methylation in individuals exposed to smoking and diagnosed with different skin cancers could also provide insights into how smoking influences cancer development.

## Materials and methods

### Overview

Our analyses used multiple genetic variants as instrumental variables that captured three different measures of smoking exposure. The first was a measure of “smoking initiation” which was generated as a binary trait based on whether participants reported ever versus never being a regular smoker^15^. The second measure was “smoking intensity,” which captures the average number of cigarettes smoked per day by ever smokers. The third measure was the LSI. The LSI is a comprehensive measure of smoking exposure that goes beyond pack-years, incorporating all smoking-related dimensions reported by study participants and was generated following the method outlined by Leffondré^30^. The score incorporates smoking intensity, smoking duration, time since cessation and a half-life constant which measures the exponentially decreasing effect of smoking at a given time on health outcomes^16^. The LSI was estimated based on following equation: LSI = (1–0.5^dur*/τ^) (0.5^tsc*/τ^) ln(int + 1), where dur = duration of smoking, τ = half-life, int = intensity (cigarettes per day), tsc = time since cessation. The value of half-life was set to 18 as it has been shown by a previous simulation study to optimize the model fit^16^. The values of LSI are expressed per one standard deviation (SD) increment. In the UK Biobank, for example, one standard deviation (SD) increment in LSI is equivalent to an individual smoking 20 cigarettes a day for 15 years and stopping 17 years ago. Full details on the LSI derivation and assumptions have been described previously^16^.

### Selection of genetic variants associated with smoking exposure

Genetic variants for smoking initiation and intensity were selected from the GWAS and Sequencing Consortium of Alcohol and Nicotine use (GSCAN); (n = 1,232,091 individuals for the smoking initiation GWAS, and n = 337,334 ever-smokers for the smoking intensity GWAS)^15^. A total of 378 and 55 conditionally independent SNPs associated with smoking initiation and intensity, respectively, at the genome-wide significance threshold (*p* < 5 × 10^−8^) were extracted from those GWASs. In a previous study, an instrumental variable constructed from smoking initiation variants was shown to predict smoking-related cancers such as lung, esophageal, stomach, cervix, and bladder^17^, indicating that the variants for smoking are valid instrument to predict smoking behaviour. In total, 126 conditionally independent variants associated with LSI were obtained from a GWAS of LSI (n = 462,690) conducted in the UK Biobank^16^. Independent SNPs were those reaching genome-wide significance level (*p*-value < 5 × 10^−8^), had a collinearity r2 < 0.001 with other lead SNPs, and were more than 10,000 Kb from other lead SNPs. The selected genetic variants accounted for 2%, 1.1% and 0.4% of the variance in smoking initiation, smoking intensity, and LSI, respectively.

### GWAS summary data for melanoma, BCC and cSCC

The melanoma GWAS meta-analysis data are from the largest melanoma GWAS to date, with up to 36,760 melanoma cases and 375,188 controls^18^. However, it includes samples from UK Biobank and 23andMe that partially overlap with the smoking initiation GWAS. To ensure independence between samples, we performed an additional melanoma GWAS meta-analysis after excluding data from the UK Biobank and 23andMe. Briefly, a fixed-effects inverse variance-weighted meta-analysis was performed using PLINK v.1.90b5.4^31^ after removing the clinically-confirmed and self-report melanoma GWAS results from the UK Biobank, and the self-reported melanoma GWAS from 23andMe. The final sample size was 26,635 cases and 67,419 controls. All included GWAS were otherwise as reported in Landi et al.^18^.

For BCC and cSCC, we obtained the GWAS summary data from the independent FinnGen cohort which comprises 14,493 BCC cases and 248,495 controls and 2,334 cSCC cases and 246,161 controls. Detailed information describing the participating study, genotyping protocol, and quality control for the FinnGen cohort has been previously provided^32^.

### Validation of instrument variables in the QSkin cohort

Before performing the risk analyses, we tested the validity of genetic instruments for smoking measures in an independent data set, the QSkin Sun and Health study. Briefly, the QSkin cohort is a population-based cohort of 43,794 participants aged between 40 and 69 years, who were randomly recruited from the Queensland population in 2011^33^. In the QSkin cohort, information on smoking measures was self-reported but a validation study showed almost perfect repeatability for smoking status (weighted kappa = 0.97, 95% CI 0.92–1.00) and other smoking measures^34^. Further details of participant recruitment, other study characteristics, and genotyping has been reported elsewhere^35,36^. Using the genetic variants for each measure of smoking, we calculated the genome-wide polygenic risk scores (PRS) for each participant in the QSkin cohort who consented and provided a DNA sample (n = 17,646). Using the—score function in Plink V1.90b6.6.23, we calculated the PRS for all three measures of smoking for each participant in the QSkin cohort by taking the sum of an individual’s risk alleles, weighted by each risk allele’s effect size^37^. We then regressed the PRS against the measured smoking parameters to assess the validity of the smoking instruments.

### Mendelian randomization analyses

Mendelian randomization (MR) is a statistical technique used in epidemiology to explore the causal relationship between an exposure, such as a risk factor or environmental factor, and an outcome, such as a disease or health condition, using observational data. MR uses genetic variants, typically SNPs, that are strongly associated with the exposure of interest as instrumental variables to estimate causal effects. This is based on the principle that genetic variants are randomly allocated at conception and are not influenced by behavioral or environmental factors. For MR to be valid, it must satisfy three core instrumental variable assumptions: (1) The genetic variant used as an instrument for the exposure is associated with the exposure (the relevance assumption); (2) The genetic variant is independent of any confounders that can bias the relationship between the exposure and the outcome (the independence assumption); and (3) The genetic variant affects the outcome only through the exposure and not through any other pathways (the exclusion restriction assumption)^38^.

We used the two-sample MR method to evaluate whether genetically predicted smoking behaviors relate to skin cancer risk. The two-sample MR, in which the variant-exposure and variant-outcome associations are measured in separate samples, offers high statistical power by combining data from multiple large consortia. For each measure of smoking behavior, individual effect estimates for each SNP were calculated using the Wald-type ratio estimator by dividing the variant-outcome association by the variant-exposure association^39^. Multiple SNPs were then combined into a multi-allelic instrument using the random-effects inverse-variance weighted (IVW) meta-analysis method^40^. We then performed sensitivity analyses (MR-Egger, weighted median, weighted and simple mode) to account for potential unbalanced horizontal pleiotropy and to evaluate whether the MR assumptions were violated^41^. We further used the MR pleiotropy residual sum and outlier test (MR-PRESSO) method to detect and correct for outlying variants^42^. We used the R software package *TwoSampleMR* for MR analyses^43^.

### Ethics approval

The QSkin study was approved by the Human Research Ethics Committee of the QIMR Berghofer Medical Research Institute (P1309; P2034), and each participant provided written informed consent to take part.

### Supplementary Information

Supplementary Information.

## Data Availability

The GWAS summary statistics for smoking initiation and intensity are available from the Sequencing Consortium of Alcohol and Nicotine use (GSCAN) (https://conservancy.umn.edu/handle/11299/201564). Lifetime smoking GWAS summary data are available for download at https://doi.org/10.5523/bris.10i96zb8gm0j81yz0q6ztei23d. All GWAS summary statistics for BCC and cSCC are publicly available at FinnGen study website (www.finngen.fi/en/access_results). The GWAS summary statistics for melanoma can be obtained via a direct request to the Melanoma Genetics Consortium (GenoMEL) at https://genomel.org/. The raw genetic and phenotypic QSkin data can be obtained by application to QSkin Principal Investigator David Whiteman at David.Whiteman@qimrberghofer.edu.au).
