# Supplementary material for: Genetic variants for smoking behaviour and risk of skin cancer
Source: Sci Rep. 2023 Oct 6;13:16873. doi: 10.1038/s41598-023-44144-0 (PMC10558453; doi:10.1038/s41598-023-44144-0)
Supplement: Supplementary file 1 — Supplementary Information. [file 41598_2023_44144_MOESM1_ESM.pdf]

Genetic variants for smoking behaviour and risk of skin cancer

Jean Claude Dusingize[1], Matthew H. Law[1,2,3], Mathias Seviiri[1,2], Catherine M. Olsen[1,3], Nirmala Pandeya[1,3], Maria Teresa Landi[4], Mark M. Iles[5], Rachel E. Neale[1,3], Jue-Sheng Ong[1], Stuart MacGregor[1,3], *David C. Whiteman[1,3]

[1]Departments of Population Health and Computational Biology, QIMR Berghofer Medical Research Institute, Brisbane, Queensland, Australia

[2]School of Biomedical Sciences, Faculty of Health, Queensland University of Technology, Brisbane, Queensland, Australia

[3] Faculty of Medicine, The University of Queensland, Queensland, Australia

[4]Division of Cancer Epidemiology and Genetics, National Cancer Institute, National Institutes of Health, Bethesda, MD, USA

[5]Leeds Institute for Data Analytics, University of Leeds, Leeds, UK

Supplementary material

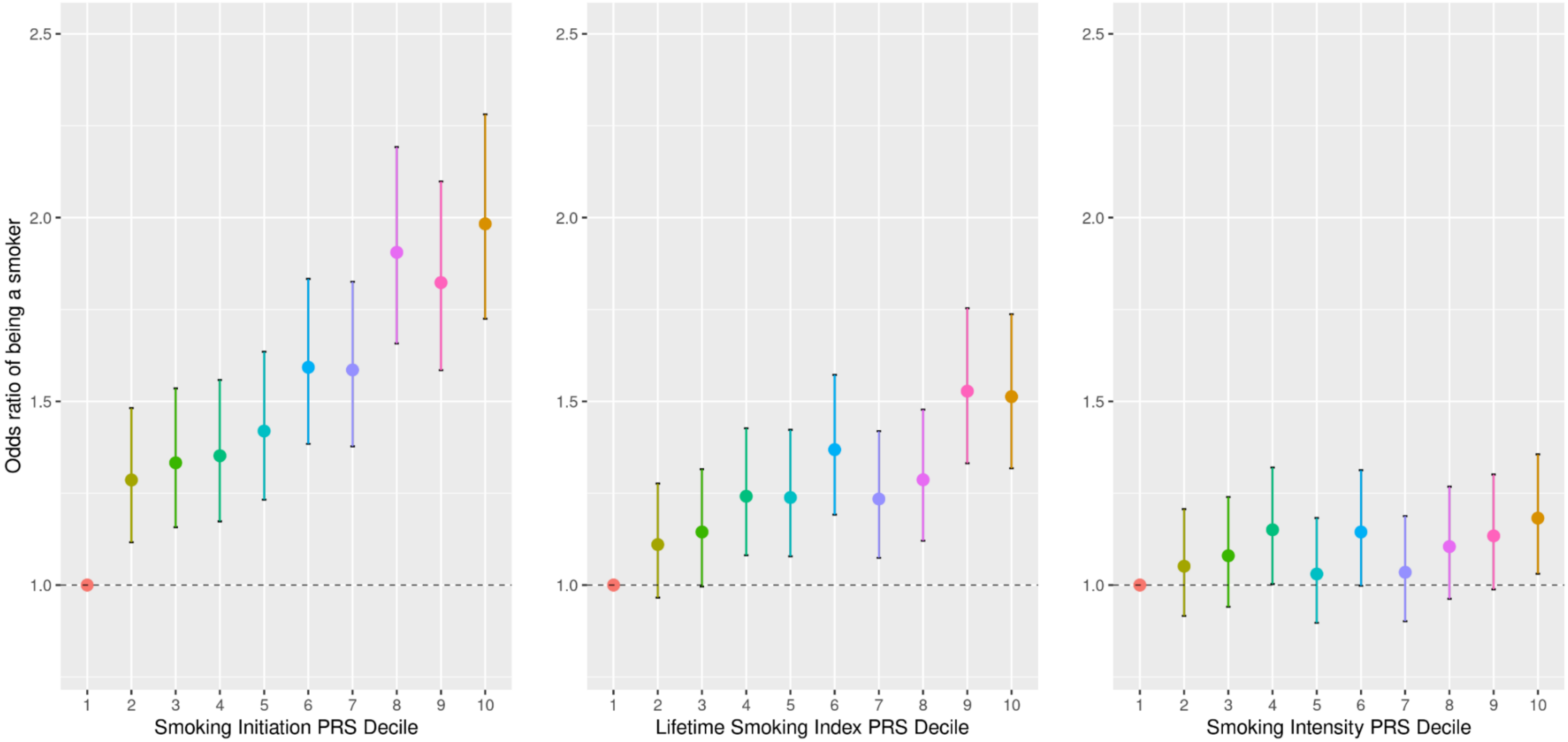

Supplementary Figure S1. The odds ratio of being a smoker in each polygenic risk score (PRS) decile of smoking initiation, intensity and lifetime amount in the QSkin cohort.

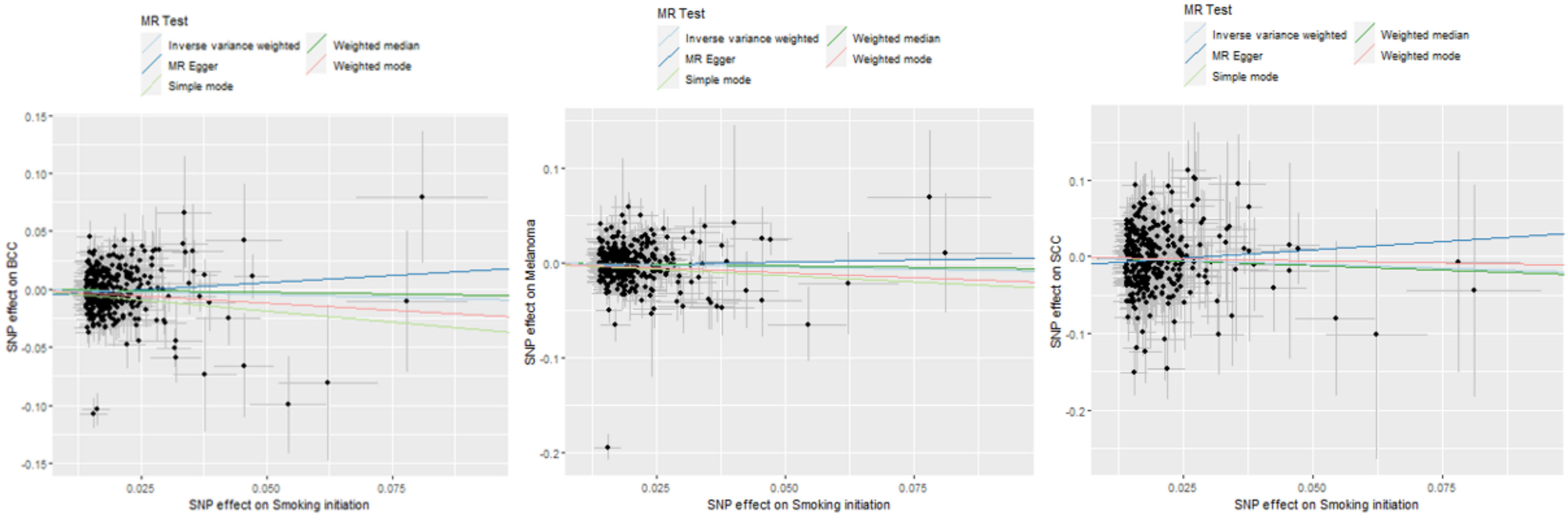

Supplementary Figure S2: Scatter plots of individual SNP-smoking initiation and SNP-basal cell carcinoma (BCC), SNP-melanoma and SNP-squamous cell carcinoma (SCC) associations. The x-axis shows the estimates of each individual genetic variants on smoking initiation, the y-axis shows the estimates (log odds ratios) of the effect of the same variants on BCC, melanoma or SCC.

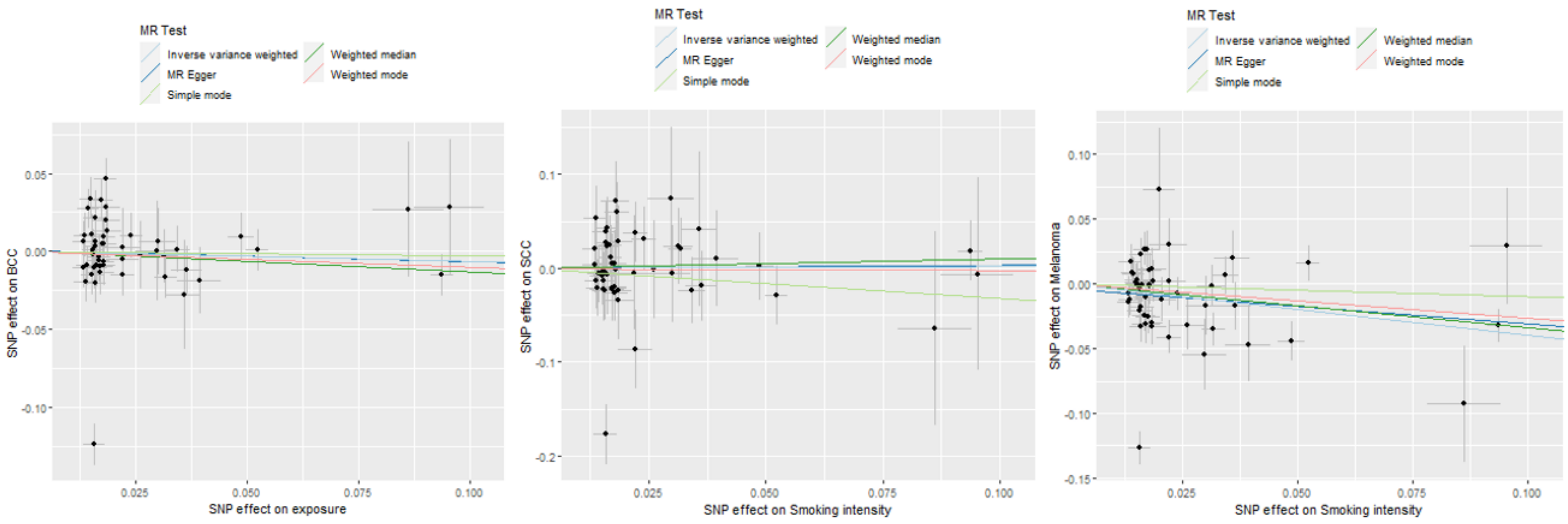

Supplementary Figure S3: Scatter plots of individual SNP-smoking intensity and SNP-basal cell carcinoma (BCC), SNP-squamous cell carcinoma (SCC), and SNP-melanoma associations. The x-axis shows the estimates of each individual genetic variants on smoking intensity, the y-axis shows the estimates (log odds ratios) of the effect of the same variants on BCC, SCC or melanoma.

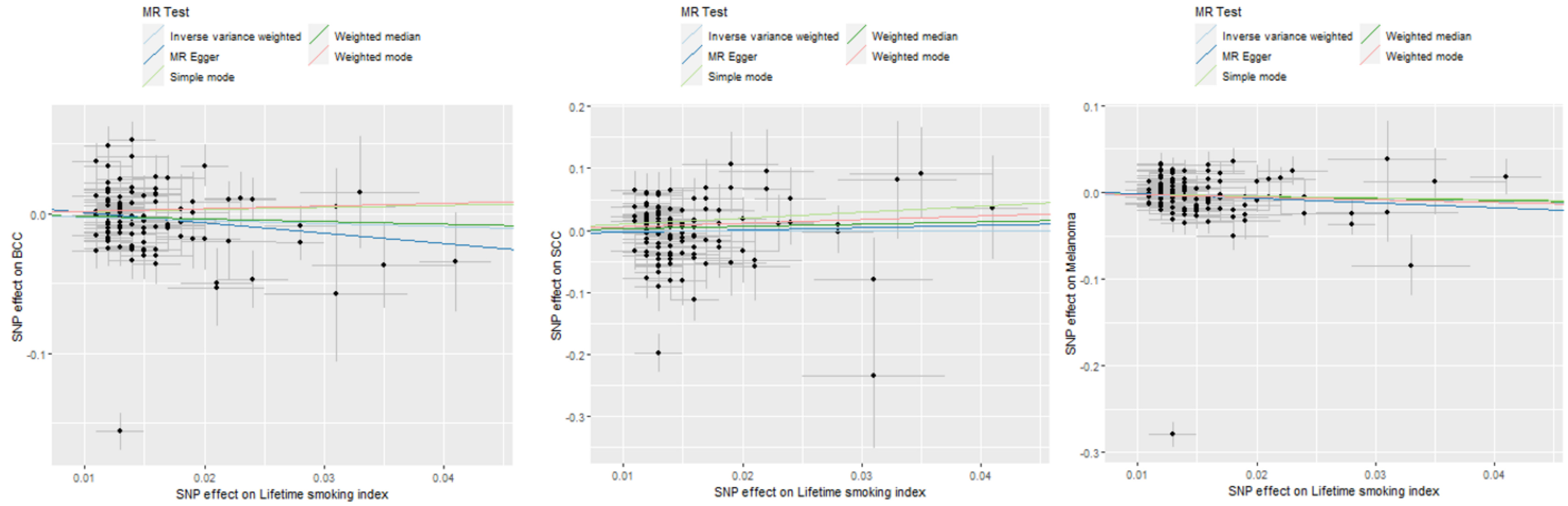

Supplementary Figure S4: Scatter plots of individual SNP-lifetime smoking index (LSI) and SNP-basal cell carcinoma (BCC), SNP-squamous cell carcinoma (SCC), and SNP-melanoma associations. The x-axis shows the estimates of each individual genetic variants on LSI, the y-axis shows the estimates (log odds ratios) of the effect of the same variants on BCC, SCC or melanoma.

Supplementary Table S1: Assessing directional pleiotropy through MR Egger intercept.

Outcome	Exposure	MR Egger intercept	P-value
**BCC**	Smoking initiation	-0.0066	0.12
	Smoking intensity	0.0002	0.97
	Lifetime smoking index	0.0079	0.40
**SCC**	Smoking initiation	-0.0125	0.14
	Smoking intensity	-0.0003	0.97
	Lifetime smoking index	-0.0059	0.72
**Melanoma**	Smoking initiation	-0.0035	0.40
	Smoking intensity	-0.0040	0.55
	Lifetime smoking index	0.0041	0.71

Supplementary Table S2. Statistical power of the study for detecting different odds ratios

Cancer type	Variance explained (%)[a]	Sample size	Proportion of cases	OR[b] 1.20	OR[b] 1.15	OR[b] 1.10	OR[b] 1.05
**Smoking initiation**							
**BCC**	2%	262,988	0.06	0.93	0.73	0.40	0.14
**SCC**	2%	248,495	0.01	0.29	0.18	0.11	0.06
**Melanoma**	2%	94,054	0.28	0.96	0.80	0.47	0.16
**Smoking intensity**							
**BCC**	1.1%	262,988	0.06	0.71	0.48	0.25	0.10
**SCC**	1.1%	248,495	0.01	0.18	0.12	0.08	0.06
**Melanoma**	1.1%	94,054	0.28	0.78	0.55	0.29	0.11
**LSI**							
**BCC**	0.4%	262,988	0.06	0.33	0.21	0.12	0.07
**SCC**	0.4%	248,495	0.01	0.10	0.08	0.06	0.05
**Melanoma**	0.4%	94,054	0.28	0.38	0.24	0.14	0.07

[a] Proportion of variance explained for the association between the SNP or allele score and the exposure variable.

[b] Assumed odds ratio per standard deviation of the exposure variable and 5 % alpha level

Power calculations were performed using the online power calculator for MR by Burgess et al. (2014)

Supplementary Table S3. A list of 55 conditionally independent SNPs associated with smoking intensity.

SNP	CHR	NEA	EA	EAF	PVALUE	BETA	SE	N
rs11264100	1	A	G	0.876	2.22E-09	-0.02217	0.003705	335394
rs2072659	1	C	G	0.099	2.51E-13	-0.02998	0.004096	335394
rs34973462	1	C	T	0.334	5.85E-09	0.015073	0.002589	335394
rs7599488	2	C	T	0.437	8.95E-09	0.014121	0.002454	337334
rs78408772	2	C	T	0.102	4.51E-08	-0.022	0.004023	337334
rs10204824	2	A	G	0.639	1.35E-12	-0.01798	0.002535	337334
rs2084533	3	C	T	0.321	6.53E-10	0.016118	0.002608	337334
rs7431710	3	G	A	0.654	1.04E-12	-0.01829	0.002566	335553
rs2236951	3	T	C	0.2	1.59E-08	-0.01719	0.003044	337334
rs699165	3	A	G	0.745	8.09E-09	0.016119	0.002793	337334
rs28813180	3	G	A	0.498	1.95E-10	-0.0155	0.002435	337334
rs1024323	4	C	T	0.382	8.66E-09	-0.01442	0.002506	337334
rs11940255	4	G	A	0.717	2.2E-10	-0.01716	0.002703	337334
rs10454798	4	G	T	0.253	1.53E-08	0.015842	0.0028	337334
rs7766641	6	G	A	0.272	2.91E-10	-0.01728	0.002743	335553
rs215600	7	G	A	0.645	4.02E-21	-0.024	0.002544	337334
rs62447179	7	G	A	0.298	9.68E-09	-0.01527	0.002662	337334
rs2741351	8	A	C	0.826	8.8E-09	0.018476	0.003211	337334
rs73229090	8	C	A	0.112	1.14E-11	0.026211	0.00386	337334
rs13253502	8	G	A	0.407	2.31E-08	-0.01384	0.002478	337334
rs4236926	8	T	G	0.766	7.66E-33	0.034267	0.002876	337334
rs790564	8	A	C	0.729	1.24E-10	-0.01762	0.002739	337334
rs75596189	9	C	T	0.112	1.84E-20	0.0358	0.00386	337334
rs3025383	9	T	C	0.187	9.78E-24	-0.03138	0.003122	337334
rs7951365	11	T	C	0.31	1.53E-11	0.017756	0.002632	337334
rs10742683	11	G	A	0.415	4.83E-08	-0.01349	0.002471	337334
rs113001570	11	A	T	0.067	1.04E-09	0.029801	0.00488	337334
rs7125588	11	A	G	0.429	6.5E-12	-0.0169	0.00246	337334
rs11846838	14	G	A	0.327	5.03E-09	0.015177	0.002595	337334
rs1115019	15	T	C	0.79	2.27E-09	-0.01786	0.002989	337334
rs632811	15	A	G	0.33	1.67E-10	-0.01775	0.002779	292829
rs4886550	15	A	G	0.288	4.58E-09	-0.01993	0.003398	211153
rs12438181	15	G	A	0.218	4.97E-10	-0.01853	0.002978	330721
rs10519203	15	G	A	0.655	3.1E-286	-0.09362	0.002587	330721
rs28438420	15	A	T	0.554	1.25E-12	0.017561	0.002474	330721
rs72740955	15	C	T	0.337	2.42E-34	0.031752	0.002601	330721
rs146009840	15	A	T	0.335	2E-17	0.02212	0.002605	330721
rs28681284	15	C	T	0.21	2.1E-58	-0.04868	0.003019	330721
rs8040868	15	T	C	0.4	1.79E-10	0.016012	0.00251	330721
rs3743063	15	A	C	0.562	1.53E-11	-0.01672	0.002478	330721

rs182317	15	G	T	0.355	1.31E-09	-0.01559	0.00257	330721
rs1592485	16	C	A	0.611	1.11E-10	-0.01615	0.002504	335394
rs12924872	16	C	T	0.463	4.39E-08	-0.01341	0.002449	335394
rs258321	16	A	G	0.429	1.53E-10	0.015796	0.002467	335394
rs4144686	18	G	A	0.167	1.35E-08	-0.01855	0.003264	337334
rs4485470	18	G	A	0.592	7.05E-10	-0.01527	0.002477	337334
rs59208569	19	G	C	0.829	2.45E-10	0.020476	0.003234	337334
rs143200968	19	G	C	0.025	6.97E-28	-0.0861	0.00786	330721
rs56113850	19	T	C	0.555	4E-99	0.05231	0.002474	330721
rs8192726	19	C	A	0.068	8.35E-16	-0.03934	0.004888	330721
rs117824460	19	A	G	0.026	7.66E-35	-0.09526	0.007727	330721
rs6078373	20	G	A	0.402	9.4E-11	0.016073	0.002483	337334
rs1737894	20	C	G	0.408	9.9E-12	0.016856	0.002477	337334
rs2273500	20	T	C	0.147	3.49E-26	0.036386	0.003438	337334
rs7281463	21	A	C	0.413	3.15E-08	0.013678	0.002473	337334

Supplementary Table S4. A list of 378 conditionally independent SNPs associated with smoking initiation.

SNP	CHR	NEA	EA	EAF	PVALUE	BETA	SE	N
**rs12130857**	1	G	A	0.324968	3.65E-11	-0.018	0.00272	1232091
**rs301807**	1	A	G	0.57	2.5E-12	0.018014	0.00257	1232091
**rs3820277**	1	G	T	0.526	1.57E-13	-0.01884	0.00255	1232091
**rs1889571**	1	T	G	0.131	4.19E-09	0.02218	0.00378	1232091
**rs10914684**	1	G	A	0.324	6.32E-09	-0.0158	0.00272	1232091
**rs2637869**	1	G	A	0.297	6.54E-11	0.01822	0.00279	1232091
**rs12755632**	1	A	G	0.316	1.93E-08	-0.01541	0.00274	1232091
**rs951740**	1	G	A	0.625	3.82E-29	0.029541	0.00263	1232091
**rs925524**	1	A	G	0.71	2.94E-08	0.015557	0.00281	1232091
**rs12022778**	1	A	C	0.202	3.18E-17	0.026825	0.00318	1227673
**rs11587399**	1	A	T	0.221	7.25E-09	-0.0178	0.00308	1227673
**rs4912332**	1	C	T	0.491	2.94E-08	0.014121	0.00255	1232091
**rs1937443**	1	C	G	0.563	1.79E-15	0.020436	0.00257	1232091
**rs1022528**	1	G	A	0.344	8.48E-11	0.017402	0.00268	1232091
**rs12740789**	1	G	A	0.178	1.18E-17	-0.0285	0.00333	1232091
**rs80054503**	1	T	C	0.116	3.1E-09	-0.02414	0.00407	1174994
**rs10789369**	1	A	G	0.615	3.39E-19	-0.02345	0.00262	1232091
**rs1514176**	1	G	A	0.58	7.67E-14	-0.0193	0.00258	1232091
**rs10873871**	1	A	G	0.207	2.82E-08	0.017452	0.00314	1232091
**rs11162019**	1	C	T	0.363	5.06E-09	-0.01549	0.00265	1232091
**rs1008078**	1	C	T	0.402	1.63E-18	0.022817	0.0026	1232091
**rs1935571**	1	T	G	0.48	6.99E-10	-0.01572	0.00255	1232091
**rs12027999**	1	T	C	0.12	5.33E-10	-0.02436	0.00392	1232091

**rs45444697**	1	C	G	0.212	2.72E-10	0.01969	0.00312	1232091
**rs2901785**	1	G	A	0.446	1.47E-11	-0.01731	0.00256	1232091
**rs147052174**	1	G	T	0.0171	2.3E-10	0.062309	0.00983	1232091
**rs35656245**	1	G	A	0.276	2.23E-08	0.015946	0.00285	1232091
**rs12739243**	1	T	C	0.221	4.45E-12	-0.02125	0.00307	1232091
**rs12563365**	1	G	A	0.556	1.05E-10	0.016559	0.00256	1232091
**rs876793**	1	T	C	0.349262	5.69E-11	-0.01792	0.00274	1174994
**rs114976176**	2	A	C	0.351571	6.04E-09	-0.01551	0.00267	1232091
**rs62106258**	2	T	C	0.047329	3.33E-14	-0.0455	0.006	1232091
**rs6731872**	2	T	G	0.826	5.35E-21	0.031598	0.00336	1232091
**rs1022376**	2	T	C	0.515821	1.66E-08	-0.01474	0.00261	1174994
**rs61533748**	2	T	C	0.384	2.82E-11	0.017436	0.00262	1232091
**rs72790288**	2	G	A	0.0282	3.28E-09	-0.04553	0.0077	1232091
**rs2710634**	2	T	C	0.521	3.36E-12	-0.01776	0.00255	1232091
**rs62137126**	2	A	G	0.121094	1.31E-09	-0.02369	0.00391	1232091
**rs1004787**	2	G	A	0.552	1.11E-28	0.028414	0.00256	1232091
**rs7598402**	2	C	G	0.492084	7.38E-09	-0.01473	0.00255	1232091
**rs10490159**	2	C	T	0.394	3.86E-11	0.017237	0.00261	1232091
**rs1518393**	2	A	C	0.619	1.3E-10	0.01686	0.00262	1232091
**rs17616642**	2	A	G	0.24687	2.1E-08	-0.01656	0.00295	1232091
**rs6730325**	2	G	A	0.609782	2.1E-08	-0.01464	0.00261	1232091
**rs2539706**	2	G	A	0.529947	1.95E-10	0.016245	0.00255	1232091
**rs7585579**	2	C	G	0.499	5.48E-15	0.020396	0.00261	1174994
**rs1863161**	2	G	A	0.56094	2.34E-09	0.015339	0.00257	1232091
**rs359247**	2	A	T	0.638652	9.89E-17	0.02203	0.00265	1232091
**rs62180324**	2	G	A	0.212	3.91E-10	-0.01952	0.00312	1232091
**rs6750107**	2	G	A	0.386875	2.6E-08	0.014565	0.00262	1232091
**rs12714017**	2	T	C	0.511	3.65E-09	0.015396	0.00261	1174994
**rs56208390**	2	A	G	0.123	2.68E-08	0.021564	0.00388	1232091
**rs11692435**	2	G	A	0.0848	4.47E-08	0.025053	0.00458	1227673
**rs13392222**	2	A	C	0.139	1.93E-10	-0.02344	0.00368	1232091
**rs1901477**	2	A	G	0.511	2.07E-31	0.030437	0.00261	1174994
**rs11889814**	2	A	C	0.128	3.44E-08	-0.02103	0.00381	1232091
**rs3811038**	2	T	C	0.279	1.58E-11	0.01914	0.00284	1232091
**rs75210106**	2	C	T	0.176676	2.33E-08	-0.01866	0.00334	1232091
**rs34399632**	2	A	G	0.232	1.46E-10	0.01935	0.00302	1232091
**rs74697736**	2	G	A	0.287239	2.43E-15	0.022296	0.00282	1232091
**rs6756212**	2	C	T	0.535	3.49E-40	-0.03389	0.00255	1232091
**rs3076896**	2	G	A	0.38988	1.99E-16	0.022646	0.00275	1108278
**rs16826827**	2	T	C	0.124	9.17E-09	-0.02221	0.00387	1232091
**rs1445649**	2	T	C	0.538	8.48E-16	0.020572	0.00256	1232091
**rs1722666**	2	C	T	0.732	2.17E-08	0.016093	0.00288	1232091

**rs11678980**	2	G	A	0.45	5.19E-12	0.017669	0.00256	1232091
**rs12474587**	2	G	T	0.429	4.83E-21	0.024231	0.00257	1232091
**rs357304**	2	T	C	0.727	5.4E-09	0.016676	0.00286	1232091
**rs13007361**	2	G	A	0.208	2.29E-08	0.017534	0.00314	1232091
**rs7600835**	2	G	A	0.342	1.8E-08	-0.01512	0.00269	1232091
**rs6750529**	2	C	T	0.744	9.26E-12	0.019907	0.00292	1232091
**rs17229285**	2	C	T	0.505	1.27E-09	-0.01548	0.00255	1232091
**rs3115418**	2	T	C	0.454	2.79E-08	-0.01422	0.00256	1232091
**rs62193862**	2	G	A	0.0999	1.99E-08	0.023846	0.00425	1232091
**rs4674916**	2	C	A	0.327671	3.06E-11	-0.01803	0.00271	1232091
**rs4674993**	2	A	G	0.2	4.85E-14	-0.02401	0.00319	1232091
**rs11713899**	3	A	C	0.171	3.15E-08	0.018719	0.00338	1232091
**rs748832**	3	A	G	0.371	6.6E-11	0.017214	0.00264	1232091
**rs10446419**	3	A	G	0.207	5.05E-10	-0.01956	0.00314	1232091
**rs13319205**	3	T	A	0.29	3.77E-09	0.01654	0.00281	1232091
**rs3172494**	3	G	T	0.115	3.4E-13	-0.02913	0.004	1227673
**rs2526390**	3	C	T	0.334	3.62E-14	0.020466	0.0027	1232091
**rs2276825**	3	T	C	0.245	1.89E-10	0.018876	0.00296	1232091
**rs2306866**	3	A	T	0.614	1.89E-10	-0.01668	0.00262	1232091
**rs73831818**	3	A	G	0.057	5.46E-09	0.032043	0.0055	1232091
**rs1910236**	3	G	A	0.469	9.91E-09	0.014644	0.00255	1232091
**rs7640107**	3	C	T	0.430789	3.46E-08	-0.01419	0.00257	1232091
**rs2734390**	3	A	G	0.372	2.09E-08	0.014771	0.00264	1232091
**rs221988**	3	A	C	0.384	1.43E-08	-0.01487	0.00262	1232091
**rs2196356**	3	G	C	0.288886	2.45E-11	-0.01877	0.00281	1232091
**rs11128203**	3	T	A	0.53	1.29E-15	0.020406	0.00255	1232091
**rs62246017**	3	G	A	0.322639	3.03E-09	-0.01617	0.00273	1232091
**rs4543050**	3	A	T	0.816	1.45E-11	0.022204	0.00329	1232091
**rs6782116**	3	C	T	0.415	1.46E-08	-0.01465	0.00259	1232091
**rs13066050**	3	C	T	0.208	1.93E-09	0.018834	0.00314	1232091
**rs12633090**	3	G	C	0.182	3.16E-12	-0.02302	0.0033	1232091
**rs1549979**	3	C	T	0.615	8.8E-21	-0.02452	0.00262	1227673
**rs74664784**	3	T	C	0.376	9.34E-13	-0.01991	0.00279	1096594
**rs57153235**	3	T	G	0.318	1.56E-12	-0.01938	0.00274	1227673
**rs6437769**	3	C	T	0.581	3.74E-08	0.014214	0.00258	1232091
**rs9288999**	3	G	A	0.735	1.5E-09	0.017441	0.00289	1232091
**rs6438436**	3	C	T	0.816	5.33E-14	0.024737	0.00329	1232091
**rs12053870**	3	T	G	0.541511	1.02E-09	0.015616	0.00256	1232091
**rs9826984**	3	G	A	0.542	3.87E-08	-0.01405	0.00256	1232091
**rs2279829**	3	C	T	0.216	2.05E-08	-0.01738	0.0031	1232091
**rs2319545**	3	C	A	0.149099	8.3E-11	0.023237	0.00358	1232091
**rs10935779**	3	C	T	0.415	2.95E-08	-0.01433	0.00259	1232091

**rs963354**	3	C	A	0.687	4.21E-08	0.015049	0.00275	1232091
**rs1714521**	3	A	C	0.411	3.07E-10	-0.0163	0.00259	1232091
**rs1449012**	3	C	T	0.463	1.77E-09	-0.01537	0.00256	1232091
**rs9850597**	3	G	A	0.816	1.65E-08	-0.01857	0.00329	1232091
**rs1187820**	3	C	T	0.439	2.69E-08	-0.01427	0.00257	1232091
**rs16828799**	3	G	T	0.156	1.83E-08	0.019769	0.00351	1232091
**rs9841807**	3	C	T	0.273	1.35E-08	0.016253	0.00286	1232091
**rs7631379**	3	T	C	0.206	3.94E-11	0.020801	0.00315	1232091
**rs4140932**	4	T	A	0.431	4.89E-08	-0.01404	0.00257	1232091
**rs12642744**	4	G	T	0.744	2.82E-08	-0.01659	0.00299	1174994
**rs59537158**	4	C	T	0.214	4.62E-13	0.022487	0.00311	1232091
**rs1389171**	4	T	A	0.241	4.45E-09	-0.01747	0.00298	1232091
**rs55944129**	4	T	C	0.267	1.06E-09	-0.01757	0.00288	1232091
**rs58400863**	4	G	A	0.347	4.89E-14	-0.02017	0.00268	1232091
**rs7657022**	4	A	G	0.489	7.34E-13	0.018291	0.00255	1232091
**rs55900829**	4	A	T	0.334806	5.63E-12	0.019132	0.00278	1165375
**rs112725451**	4	C	T	0.169	1.65E-14	0.026092	0.0034	1232091
**rs1160685**	4	C	G	0.45	2.31E-09	0.015302	0.00256	1232091
**rs1435479**	4	G	T	0.28748	5.68E-09	0.01639	0.00282	1232091
**rs3934797**	4	G	A	0.182	1.12E-10	-0.0213	0.0033	1232091
**rs71602617**	4	C	T	0.216	2.1E-08	-0.01777	0.00317	1174994
**rs7696257**	4	G	A	0.366	6.78E-09	0.015331	0.00264	1232091
**rs13109980**	4	G	A	0.326	3.37E-16	-0.02218	0.00272	1232091
**rs1116690**	4	A	G	0.742	2.16E-08	0.016291	0.00291	1232091
**rs13110073**	4	T	C	0.395	3.24E-21	-0.02464	0.00261	1232091
**rs28717373**	4	C	T	0.356165	6.16E-10	-0.01647	0.00266	1232091
**rs62340589**	4	G	C	0.201	4.31E-08	0.017413	0.00318	1232091
**rs12517438**	5	T	G	0.538	1.89E-09	0.015354	0.00256	1232091
**rs35375873**	5	G	C	0.11	3.29E-11	-0.02701	0.00407	1232091
**rs986714**	5	A	T	0.445	4.13E-10	-0.01603	0.00256	1232091
**rs71592686**	5	T	C	0.274	3.85E-13	0.020737	0.00286	1232091
**rs2028269**	5	G	A	0.399	5.19E-10	0.016165	0.0026	1232091
**rs6874731**	5	T	G	0.484	1.83E-09	0.015318	0.00255	1232091
**rs6452785**	5	C	T	0.474	4.69E-26	-0.02688	0.00255	1232091
**rs10805858**	5	A	T	0.335286	1.88E-11	0.018124	0.0027	1232091
**rs181508347**	5	T	G	0.00965	4.95E-10	0.081076	0.01303	1232091
**rs42417**	5	C	T	0.691	8.27E-10	0.01693	0.00276	1232091
**rs72780746**	5	T	C	0.173	2.05E-14	-0.02576	0.00337	1232091
**rs10060196**	5	C	A	0.580606	1.29E-12	0.018312	0.00258	1232091
**rs72789626**	5	T	A	0.136	5.13E-12	-0.02564	0.00372	1232091
**rs17165769**	5	A	G	0.394872	9.56E-10	0.01594	0.00261	1232091
**rs329124**	5	A	G	0.428	1.96E-10	-0.01639	0.00257	1232091

**rs1385108**	5	C	T	0.239	3.84E-10	0.018704	0.00299	1232091
**rs1173461**	5	C	T	0.327	9.51E-10	0.016609	0.00272	1232091
**rs11956866**	5	T	G	0.567	7.82E-09	-0.01484	0.00257	1232091
**rs3909281**	5	T	G	0.536	1.62E-16	0.021067	0.00255	1232091
**rs3843905**	5	C	T	0.403	5.41E-09	-0.01515	0.0026	1232091
**rs79476395**	5	A	G	0.0726	1.04E-11	0.033374	0.00491	1232091
**rs6890961**	5	C	T	0.624	2.13E-13	-0.01931	0.00263	1232091
**rs4044321**	5	A	G	0.644	1.75E-17	-0.02264	0.00266	1232091
**rs2173019**	5	T	A	0.177	2.98E-17	0.028207	0.00334	1232091
**rs10042827**	5	T	C	0.681	9.41E-10	0.016717	0.00273	1232091
**rs359431**	5	C	T	0.56	3.16E-08	-0.0142	0.00257	1232091
**rs1059490**	6	T	C	0.367	2.16E-12	-0.01859	0.00265	1227673
**rs6932350**	6	T	A	0.454655	5.13E-09	0.014968	0.00256	1227673
**rs1150668**	6	T	G	0.419	8.54E-13	-0.01851	0.00259	1227673
**rs1632941**	6	T	C	0.46	6.67E-10	-0.01581	0.00256	1227673
**rs3218116**	6	C	T	0.256	1.05E-11	-0.01984	0.00292	1232091
**rs160631**	6	T	G	0.731	1.87E-09	-0.01726	0.00287	1232091
**rs7743165**	6	T	G	0.495	4.15E-14	0.019256	0.00255	1232091
**rs79180767**	6	C	T	0.253	7E-12	0.020092	0.00293	1232091
**rs10945141**	6	G	A	0.263	3.59E-10	0.018142	0.00289	1232091
**rs17554906**	6	G	C	0.444	3.14E-08	0.014185	0.00256	1232091
**rs619087**	6	A	G	0.422	3.1E-08	0.01427	0.00258	1232091
**rs6568832**	6	G	A	0.753851	1.74E-10	0.018869	0.00296	1232091
**rs12195240**	6	G	A	0.285	1.08E-18	0.024911	0.00282	1232091
**rs6936160**	6	C	T	0.698	4.2E-13	0.020107	0.00277	1232091
**rs12530388**	6	A	C	0.511	5.83E-13	-0.01836	0.00255	1232091
**rs3800227**	6	A	G	0.742	3.64E-09	0.017178	0.00291	1232091
**rs118202**	6	G	T	0.812	1.9E-29	-0.03675	0.00326	1232091
**rs73008357**	6	A	C	0.121	2.44E-08	-0.02231	0.004	1174994
**rs9331343**	6	T	C	0.568	3.9E-08	-0.01413	0.00257	1232091
**rs10698713**	6	G	A	0.0544	2.38E-09	-0.03352	0.00562	1232091
**rs1737329**	6	C	G	0.742	5.08E-09	0.017029	0.00291	1232091
**rs10272990**	7	T	C	0.327622	1.27E-14	-0.02092	0.00271	1232091
**rs6948707**	7	T	G	0.419	4.24E-21	0.024347	0.00258	1232091
**rs10259715**	7	T	A	0.209918	6.42E-09	-0.01867	0.00322	1165375
**rs13237637**	7	G	C	0.485	1.54E-20	-0.02368	0.00255	1232091
**rs79631993**	7	A	C	0.216281	3.67E-08	-0.01703	0.00309	1232091
**rs7809303**	7	G	A	0.325	3.48E-15	-0.02142	0.00272	1232091
**rs7802996**	7	C	T	0.166	1.06E-09	-0.02088	0.00342	1232091
**rs1030015**	7	G	T	0.519564	2.15E-08	0.01429	0.00255	1232091
**rs4727189**	7	T	C	0.344	3E-08	0.01486	0.00268	1232091
**rs76841737**	7	C	G	0.103	3.26E-08	-0.02315	0.00419	1232091

**rs11768481**	7	C	A	0.34	5.23E-12	-0.01856	0.00269	1232091
**rs1799068**	7	G	T	0.379	2.59E-10	0.01661	0.00263	1232091
**rs13437771**	7	A	G	0.155	1.39E-14	-0.02711	0.00352	1232091
**rs11766326**	7	T	C	0.506	1.79E-11	-0.01754	0.00261	1174994
**rs6968380**	7	G	A	0.681	1.05E-17	-0.02342	0.00273	1232091
**rs112913817**	7	A	G	0.011318	9.28E-11	0.078056	0.01204	1232091
**rs10233018**	7	A	G	0.516	4.77E-22	0.024612	0.00255	1232091
**rs10953957**	7	G	A	0.386	3.66E-08	0.014406	0.00262	1232091
**rs77283305**	7	G	A	0.305819	3.91E-08	-0.0152	0.00277	1232091
**rs10279261**	7	G	A	0.618	6.05E-13	-0.01887	0.00262	1232091
**rs1561112**	7	T	C	0.412815	3.84E-09	-0.01524	0.00259	1232091
**rs2952251**	8	A	G	0.74437	4.24E-08	0.016413	0.003	1170576
**rs4326350**	8	C	G	0.493	5.16E-12	-0.01761	0.00255	1227673
**rs11780471**	8	G	A	0.063121	1.57E-13	-0.03868	0.00524	1232091
**rs11783093**	8	C	T	0.158	2.07E-41	-0.04712	0.00349	1232091
**rs1565735**	8	T	A	0.204463	1.33E-09	-0.01916	0.00316	1232091
**rs7836565**	8	C	T	0.718	4.36E-08	-0.01551	0.00283	1232091
**rs13261666**	8	G	T	0.517	4.36E-15	-0.01999	0.00255	1232091
**rs3850736**	8	C	G	0.474	6.43E-14	0.019128	0.00255	1232091
**rs2063976**	8	C	T	0.664955	7.45E-14	-0.02018	0.0027	1232091
**rs6993429**	8	C	A	0.453	9.87E-14	-0.01905	0.00256	1232091
**rs6986430**	8	T	C	0.222377	1.99E-15	-0.02434	0.00306	1232091
**rs9987376**	8	T	G	0.574251	2.01E-15	-0.02047	0.00258	1232091
**rs290601**	8	C	T	0.274	1.14E-08	0.01631	0.00286	1232091
**rs3847244**	9	C	T	0.47	2.6E-13	0.018672	0.00255	1232091
**rs11791671**	9	C	T	0.067315	4.24E-08	0.02785	0.00508	1232091
**rs7024924**	9	T	C	0.174	1.9E-08	0.018892	0.00336	1232091
**rs6474609**	9	T	A	0.586731	1.71E-09	-0.01559	0.00259	1232091
**rs1931431**	9	G	C	0.478	8.56E-13	0.018233	0.00255	1232091
**rs7867822**	9	A	G	0.673	2.76E-08	-0.0151	0.00272	1232091
**rs10966092**	9	T	C	0.267	1.12E-12	-0.02049	0.00288	1232091
**rs10969352**	9	T	A	0.5	1.82E-08	0.014347	0.00255	1232091
**rs4877285**	9	G	A	0.668249	2.1E-11	-0.01813	0.00271	1232091
**rs1930371**	9	C	T	0.241	7.09E-09	-0.01724	0.00298	1232091
**rs2378662**	9	G	A	0.541	2.67E-09	0.015212	0.00256	1232091
**rs1927901**	9	T	C	0.553	3.1E-08	-0.01418	0.00256	1232091
**rs4837631**	9	C	T	0.446	2.03E-09	-0.01536	0.00256	1232091
**rs1759433**	9	G	A	0.48	1.69E-09	0.015365	0.00255	1232091
**rs34553878**	9	A	G	0.111	1.17E-09	0.024671	0.00406	1232091
**rs7026534**	9	T	G	0.703821	2.68E-09	-0.0166	0.00279	1232091
**rs10858334**	9	C	G	0.14	1.18E-09	0.022871	0.00376	1174994
**rs10905461**	10	T	C	0.748	2.36E-08	-0.01639	0.00293	1232091

**rs7920501**	10	T	A	0.465	1.25E-09	-0.01552	0.00255	1232091
**rs1291821**	10	A	G	0.534	1.39E-08	0.014493	0.00255	1232091
**rs11258417**	10	C	T	0.391	2.71E-08	-0.01451	0.00261	1232091
**rs7072776**	10	A	G	0.712	5.66E-15	-0.02197	0.00281	1232091
**rs2796793**	10	G	A	0.452	1.55E-08	0.014481	0.00256	1232091
**rs1733760**	10	T	C	0.51	6.7E-09	0.014773	0.00255	1232091
**rs7921378**	10	G	C	0.482	6.1E-20	-0.02331	0.00255	1232091
**rs7901883**	10	G	A	0.230322	1.98E-10	-0.01926	0.00303	1232091
**rs11594623**	10	T	C	0.234241	7.45E-20	0.02744	0.00301	1232091
**rs11191269**	10	C	G	0.193294	4.61E-08	0.017643	0.00323	1232091
**rs28408682**	10	A	G	0.600019	1.41E-10	0.016673	0.0026	1232091
**rs12244388**	10	G	A	0.35	4.31E-22	0.025815	0.00267	1232091
**rs111842178**	10	A	G	0.231008	2.24E-12	0.022453	0.0032	1101012
**rs34970111**	10	C	T	0.458	1.28E-08	-0.01456	0.00256	1232091
**rs9787523**	10	T	C	0.418	1.42E-09	-0.01563	0.00258	1232091
**rs11192347**	10	G	A	0.104	6.15E-10	-0.02645	0.00427	1174994
**rs10885480**	10	T	C	0.284	3.83E-11	-0.01868	0.00283	1232091
**rs4752018**	10	C	A	0.231	4.42E-10	0.018854	0.00302	1232091
**rs9423279**	10	C	G	0.645	3.06E-12	-0.01858	0.00266	1232091
**rs6265**	11	C	T	0.188	2.81E-19	-0.02928	0.00326	1232091
**rs4275621**	11	A	G	0.382	3.76E-16	-0.02137	0.00262	1232091
**rs62618693**	11	C	T	0.0428	2.09E-08	-0.03527	0.00629	1232091
**rs2939756**	11	G	A	0.48	7.45E-10	-0.0157	0.00255	1232091
**rs1381775**	11	T	C	0.712	2.79E-08	-0.01561	0.00281	1232091
**rs2959084**	11	G	A	0.704674	9.82E-10	0.01708	0.00279	1232091
**rs3740977**	11	T	C	0.167	1.17E-08	0.019474	0.00342	1232091
**rs61886926**	11	C	T	0.384	7.3E-12	-0.01794	0.00262	1232091
**rs61884449**	11	C	T	0.149183	2.32E-08	0.019975	0.00358	1232091
**rs644740**	11	C	T	0.457	3.67E-08	-0.01408	0.00256	1232091
**rs7943721**	11	G	A	0.829	3.58E-10	-0.02121	0.00338	1232091
**rs7929518**	11	A	G	0.773	2.55E-10	0.019236	0.00304	1232091
**rs586699**	11	G	A	0.543	7.29E-09	-0.0148	0.00256	1232091
**rs76460663**	11	C	G	0.041056	4.15E-11	-0.04235	0.00642	1232091
**rs2155646**	11	T	C	0.4	9.44E-48	0.037777	0.0026	1232091
**rs78239456**	11	A	T	0.376527	9.37E-12	-0.0185	0.00271	1158109
**rs1713676**	11	A	G	0.522512	5.38E-11	-0.01673	0.00255	1232091
**rs238896**	11	G	A	0.49	3.65E-11	-0.01687	0.00255	1232091
**rs540860**	11	A	G	0.543	5.75E-12	0.017609	0.00256	1232091
**rs1944689**	11	G	T	0.785911	1.27E-08	0.01768	0.00311	1232091
**rs1834306**	11	A	G	0.579399	1.96E-08	-0.01449	0.00258	1232091
**rs1106363**	11	C	T	0.344579	9.2E-11	0.017375	0.00268	1232091
**rs2010921**	11	G	A	0.311	2.47E-10	0.017429	0.00275	1232091

**rs11057005**	12	A	G	0.441	9.12E-10	-0.01571	0.00257	1232091
**rs13906**	12	C	T	0.109	1.98E-09	-0.02453	0.00409	1232091
**rs4759229**	12	A	G	0.656	6.53E-09	0.01557	0.00268	1232091
**rs7969559**	12	A	G	0.713	1.53E-09	-0.01702	0.00282	1232091
**rs7134009**	12	T	C	0.287	4.3E-08	-0.0158	0.00288	1174994
**rs77215829**	12	A	C	0.131	2.02E-10	-0.02404	0.00378	1227673
**rs1109480**	12	G	A	0.384	1.84E-10	-0.01669	0.00262	1232091
**rs11611651**	12	G	A	0.0868	2.05E-09	0.027114	0.00453	1232091
**rs17197663**	13	G	A	0.125	2.06E-08	-0.02159	0.00385	1232091
**rs4264267**	13	C	T	0.527042	6.82E-09	0.014792	0.00255	1232091
**rs61959481**	13	G	A	0.21	7.95E-11	-0.02034	0.00313	1232091
**rs3098272**	13	A	C	0.798794	2.08E-08	-0.01781	0.00318	1232091
**rs9538162**	13	T	C	0.415874	1.76E-11	0.017379	0.00258	1232091
**rs1413119**	13	C	T	0.396318	4.77E-09	-0.01526	0.0026	1232091
**rs56367474**	13	C	T	0.304	4.2E-10	-0.0173	0.00277	1232091
**rs55786907**	13	A	G	0.162492	1.84E-08	0.019445	0.00345	1232091
**rs4886207**	13	T	C	0.637	8.78E-10	-0.01625	0.00265	1232091
**rs9540731**	13	C	T	0.509	3.42E-12	-0.01773	0.00255	1232091
**rs9545155**	13	T	C	0.478	3.04E-10	-0.01607	0.00255	1232091
**rs1772572**	13	C	A	0.324132	5.62E-10	-0.01687	0.00272	1232091
**rs75674569**	13	G	A	0.0997	2.58E-09	-0.02534	0.00425	1232091
**rs7333559**	13	G	A	0.783	5.94E-14	-0.02321	0.00309	1232091
**rs1108130**	13	T	A	0.212	1.57E-14	0.023944	0.00312	1232091
**rs12855717**	13	C	T	0.538	1.22E-09	0.015524	0.00256	1232091
**rs12878369**	14	C	A	0.414762	1.6E-11	0.017437	0.00259	1230262
**rs2145451**	14	T	C	0.193	5.44E-10	-0.02005	0.00323	1230262
**rs9323328**	14	A	G	0.537	2.55E-08	-0.01424	0.00256	1230262
**rs1811739**	14	G	A	0.248	5.97E-10	0.018272	0.00295	1230262
**rs8005334**	14	T	G	0.36	3.44E-10	0.016673	0.00266	1230262
**rs34940743**	14	A	G	0.346	2.8E-09	0.015925	0.00268	1230262
**rs2925128**	14	C	T	0.385191	3.67E-10	0.01682	0.00268	1173165
**rs1381287**	14	C	T	0.467	1.81E-12	0.018017	0.00256	1230262
**rs55913542**	14	G	T	0.175	3.25E-08	0.018562	0.00336	1230262
**rs1435672**	15	T	C	0.56	3.82E-08	0.014105	0.00257	1232091
**rs281296**	15	G	A	0.357	1.59E-20	0.024689	0.00266	1232091
**rs1435741**	15	G	A	0.432951	1.09E-12	0.018309	0.00257	1232091
**rs56902655**	15	T	G	0.136	4.09E-09	-0.02186	0.00372	1232091
**rs2289791**	15	G	T	0.247	2.01E-09	-0.01773	0.00295	1232091
**rs60833441**	15	A	G	0.461	2.28E-08	-0.01428	0.00256	1232091
**rs62007780**	15	G	T	0.416	7.48E-10	-0.01591	0.00258	1232091
**rs12442563**	15	G	T	0.223	3.13E-14	-0.02323	0.00306	1232091
**rs4310804**	15	C	G	0.247	7.55E-10	-0.01819	0.00295	1232091

**rs8027457**	15	T	C	0.511	1.88E-09	0.015314	0.00255	1232091
**rs1139897**	16	G	A	0.23	1.77E-15	-0.02409	0.00303	1232091
**rs11076962**	16	T	C	0.279	1.2E-10	0.0183	0.00284	1232091
**rs7192140**	16	T	C	0.498	3.4E-11	-0.01688	0.00255	1232091
**rs9922607**	16	C	T	0.2	3.42E-12	-0.02216	0.00319	1232091
**rs9941217**	16	C	G	0.352204	3.5E-12	-0.01856	0.00267	1232091
**rs7188873**	16	A	G	0.612998	8.46E-15	0.020296	0.00262	1232091
**rs6497840**	16	G	A	0.707	2.01E-15	0.02277	0.00287	1174994
**rs4785187**	16	G	A	0.223	6.55E-11	0.019977	0.00306	1232091
**rs8050598**	16	C	T	0.254117	1.76E-10	0.01867	0.00293	1232091
**rs12918191**	16	A	G	0.243	3.14E-11	-0.01973	0.00297	1232091
**rs9302604**	16	A	G	0.435	3.29E-13	0.01871	0.00257	1232091
**rs9936784**	16	T	G	0.53418	4.33E-08	0.013989	0.00255	1232091
**rs62052916**	16	A	T	0.0701	1.62E-10	-0.03191	0.00499	1232091
**rs4788676**	16	T	C	0.228528	4.92E-09	-0.01775	0.00303	1232091
**rs61537885**	16	T	C	0.037215	8.06E-09	-0.04006	0.00694	1158109
**rs117657830**	16	A	G	0.0417	3.18E-09	-0.03776	0.00637	1232091
**rs1050847**	16	C	T	0.559	7.37E-09	-0.01483	0.00257	1232091
**rs11642231**	16	G	A	0.369	3.44E-09	-0.0156	0.00264	1232091
**rs4790874**	17	C	T	0.532	8.43E-12	0.017449	0.00255	1232091
**rs11078713**	17	A	G	0.419348	1.59E-08	-0.01458	0.00258	1232091
**rs28441558**	17	T	C	0.0563	1.24E-10	-0.03556	0.00553	1232091
**rs11651955**	17	G	A	0.499	3.74E-08	-0.01403	0.00255	1232091
**rs67777803**	17	G	T	0.172	3.18E-13	-0.0246	0.00338	1232091
**rs2344976**	17	T	C	0.612	7.98E-09	-0.01509	0.00261	1232091
**rs3764351**	17	G	A	0.657	3.89E-08	-0.01475	0.00268	1232091
**rs72836318**	17	T	C	0.246	7E-09	-0.01712	0.00296	1232091
**rs17692129**	17	C	T	0.331	4.57E-13	0.019599	0.00271	1232091
**rs75919030**	17	T	C	0.267	3.35E-13	-0.02097	0.00288	1232091
**rs2938134**	17	C	A	0.673	3.14E-10	-0.0175	0.00278	1174994
**rs2587507**	17	T	C	0.502	8.69E-09	-0.01466	0.00255	1232091
**rs34342129**	18	T	C	0.509	2.13E-08	-0.01428	0.00255	1232091
**rs4476253**	18	G	A	0.24	5.78E-10	-0.01849	0.00298	1232091
**rs7505855**	18	C	T	0.586	5.31E-11	-0.01698	0.00259	1232091
**rs8096225**	18	A	C	0.703	2.63E-08	0.015525	0.00279	1232091
**rs67050670**	18	A	G	0.229	2.34E-11	-0.02027	0.00303	1232091
**rs2359180**	18	A	G	0.369	4.98E-08	-0.01439	0.00264	1232091
**rs72898831**	18	A	G	0.155	4.14E-12	-0.02442	0.00352	1232091
**rs8083764**	18	G	T	0.306171	7.97E-09	-0.01595	0.00276	1232091
**rs1373178**	18	T	G	0.588	4.16E-15	-0.02032	0.00259	1232091
**rs62098013**	18	G	A	0.365322	2.24E-11	0.01771	0.00265	1232091
**rs72938304**	18	G	A	0.113	1.36E-11	-0.02721	0.00402	1232091

**rs11872397**	18	G	A	0.253	5.2E-09	-0.01711	0.00293	1232091
**rs71367544**	18	C	T	0.203	8.54E-11	0.020552	0.00317	1232091
**rs76608582**	19	C	A	0.0489	4.88E-09	-0.03455	0.00591	1232091
**rs10853981**	19	G	A	0.33036	4.88E-08	0.014787	0.00271	1232091
**rs113230003**	19	G	A	0.255	1.05E-10	-0.01888	0.00292	1232091
**rs8103660**	19	T	C	0.354431	3.03E-09	0.015803	0.00266	1232091
**rs117734003**	19	G	C	0.0673	2.57E-09	0.030299	0.00509	1232091
**rs1126757**	19	C	T	0.473	2.92E-08	0.014162	0.00255	1232091
**rs6050446**	20	A	G	0.971	8.8E-13	0.05441	0.00761	1225969
**rs6058782**	20	C	T	0.908	1.78E-11	0.02971	0.00442	1225969
**rs1555445**	20	A	T	0.318	7.75E-12	0.018763	0.00274	1225969
**rs6073075**	20	T	A	0.824	2.44E-08	-0.0187	0.00335	1225969
**rs910912**	20	T	C	0.739	7.82E-09	-0.01677	0.00291	1227798
**rs6011779**	20	C	T	0.806	2.83E-09	-0.01918	0.00323	1227798
**rs3810496**	20	T	C	0.619436	1.54E-09	0.015881	0.00263	1227798
**rs4818005**	21	G	A	0.581	1.09E-14	-0.02043	0.00264	1174994
**rs139896**	22	T	C	0.648	7.14E-09	0.01544	0.00267	1232091
**rs4822102**	22	C	T	0.618	2.78E-10	-0.01654	0.00262	1232091
**rs9627272**	22	G	C	0.407	2.42E-09	-0.01547	0.00259	1232091

Supplementary Table S5. A list of 126 conditionally independent SNPs associated with lifetime smoking index (LSI).

**SNP**	**CHR**	**EA**	**NEA**	**EAF**	**BETA**	**SE**	**PVALUE**
**rs8042849**	15	C	T	0.342	0.028	0.002	1.80E-39
**rs113382419**	9	C	A	0.889	-0.041	0.003	3.00E-37
**rs6011779**	20	C	T	0.191	0.028	0.003	2.30E-27
**rs9919670**	11	G	A	0.612	-0.022	0.002	7.60E-27
**rs2890772**	2	G	T	0.413	-0.02	0.002	2.10E-22
**rs35175834**	15	G	A	0.788	-0.024	0.002	4.60E-22
**rs12244388**	10	G	A	0.661	-0.019	0.002	1.40E-19
**rs11783093**	8	C	T	0.839	0.023	0.003	1.20E-16
**rs11210229**	1	A	G	0.384	0.017	0.002	2.00E-16
**rs62155874**	2	A	G	0.873	-0.024	0.003	5.20E-16
**rs10226228**	7	A	G	0.63	-0.016	0.002	2.00E-15
**rs6119897**	20	G	A	0.762	-0.018	0.002	3.60E-15
**rs2867112**	2	T	G	0.835	0.021	0.003	4.80E-15
**rs986391**	5	G	A	0.367	0.016	0.002	9.40E-15
**rs3742365**	14	T	C	0.595	-0.016	0.002	2.50E-14
**rs2401924**	7	G	C	0.502	0.015	0.002	2.70E-14
**rs7807019**	7	A	G	0.54	-0.015	0.002	6.70E-14
**rs549845**	1	G	A	0.301	0.016	0.002	8.30E-14
**rs10922907**	1	A	T	0.451	0.015	0.002	3.00E-13

**rs7569203**	2	A	C	0.689	-0.016	0.002	7.40E-13
**rs17309874**	11	G	A	0.74	-0.016	0.002	9.70E-13
**rs6778080**	3	T	C	0.267	0.016	0.002	1.30E-12
**rs8042134**	15	T	G	0.541	-0.014	0.002	1.30E-12
**rs17576594**	4	G	A	0.724	0.016	0.002	1.70E-12
**rs7766610**	6	C	A	0.183	0.018	0.003	2.20E-12
**rs1922018**	7	C	T	0.364	0.014	0.002	3.00E-12
**rs7553348**	1	G	A	0.438	0.014	0.002	5.20E-12
**rs7528604**	1	G	A	0.566	0.014	0.002	5.70E-12
**rs329120**	5	C	T	0.581	0.014	0.002	6.30E-12
**rs12623702**	2	A	G	0.613	-0.014	0.002	7.70E-12
**rs13296519**	9	G	T	0.606	-0.014	0.002	8.10E-12
**rs6935954**	6	A	G	0.421	0.014	0.002	8.20E-12
**rs4671357**	2	T	C	0.519	-0.014	0.002	1.10E-11
**rs3896224**	10	A	G	0.585	0.014	0.002	1.10E-11
**rs326341**	3	G	A	0.525	0.014	0.002	1.20E-11
**rs4391802**	11	A	G	0.707	0.015	0.002	1.40E-11
**rs72678864**	4	G	A	0.829	0.018	0.003	1.60E-11
**rs112282219**	11	G	A	0.959	-0.033	0.005	3.80E-11
**rs10879871**	12	T	G	0.343	-0.014	0.002	5.00E-11
**rs889398**	16	C	T	0.588	0.013	0.002	6.30E-11
**rs4473348**	2	A	T	0.25	-0.015	0.002	6.40E-11
**rs1221148**	9	C	G	0.587	0.013	0.002	7.30E-11
**rs317021**	4	T	A	0.814	-0.017	0.003	1.10E-10
**rs1933270**	1	T	G	0.364	0.013	0.002	1.50E-10
**rs8614**	17	C	A	0.817	-0.017	0.003	1.80E-10
**rs11255908**	10	T	G	0.743	-0.015	0.002	2.30E-10
**rs13153393**	5	A	G	0.884	-0.02	0.003	2.50E-10
**rs2678670**	2	A	T	0.486	0.013	0.002	3.10E-10
**rs7333559**	13	G	A	0.212	0.015	0.002	3.20E-10
**rs76608582**	19	C	A	0.953	0.031	0.005	3.20E-10
**rs421983**	3	T	C	0.519	0.013	0.002	3.30E-10
**rs4543592**	9	T	C	0.52	-0.012	0.002	4.50E-10
**rs11948770**	5	T	C	0.768	-0.015	0.002	4.90E-10
**rs7039819**	9	G	A	0.427	0.013	0.002	5.10E-10
**rs10282292**	7	C	T	0.362	0.013	0.002	5.90E-10
**rs2838834**	21	C	T	0.699	-0.013	0.002	6.30E-10
**rs624833**	4	T	G	0.695	0.013	0.002	6.60E-10
**rs62135536**	2	C	T	0.968	0.035	0.006	8.00E-10
**rs3811038**	2	T	C	0.724	-0.014	0.002	8.90E-10
**rs359243**	2	T	C	0.393	-0.013	0.002	9.50E-10
**rs11768481**	7	C	A	0.666	0.013	0.002	9.90E-10

**rs6779302**	3	G	T	0.633	-0.013	0.002	1.20E-09
**rs35169606**	8	T	G	0.612	0.013	0.002	1.20E-09
**rs67596067**	17	G	A	0.649	-0.013	0.002	1.20E-09
**rs2675638**	10	G	A	0.581	0.012	0.002	1.30E-09
**rs75742406**	11	G	A	0.739	0.014	0.002	1.30E-09
**rs71367545**	18	G	A	0.791	-0.015	0.002	1.40E-09
**rs71627581**	5	G	A	0.889	0.019	0.003	1.60E-09
**rs13016665**	2	C	A	0.577	-0.012	0.002	1.80E-09
**rs369230**	16	G	T	0.308	-0.013	0.002	1.80E-09
**rs10052591**	5	T	C	0.573	0.012	0.002	2.10E-09
**rs3769949**	2	T	A	0.528	-0.012	0.002	2.50E-09
**rs7155595**	14	A	C	0.674	-0.013	0.002	2.50E-09
**rs7077678**	10	C	T	0.623	0.012	0.002	2.60E-09
**rs860326**	14	C	T	0.428	0.012	0.002	2.70E-09
**rs12202536**	6	A	G	0.513	-0.012	0.002	2.80E-09
**rs4814873**	20	C	T	0.767	0.014	0.002	2.90E-09
**rs147412694**	21	G	A	0.85	-0.017	0.003	2.90E-09
**rs9842947**	3	C	T	0.326	-0.013	0.002	3.10E-09
**rs2894808**	6	T	A	0.922	-0.022	0.004	3.50E-09
**rs12708665**	16	A	G	0.285	-0.013	0.002	3.50E-09
**rs202645**	22	A	G	0.203	-0.015	0.002	3.90E-09
**rs62098013**	18	G	A	0.64	-0.012	0.002	4.10E-09
**rs4957528**	5	A	C	0.208	-0.015	0.002	4.20E-09
**rs1246265**	9	T	C	0.305	-0.013	0.002	4.20E-09
**rs6598539**	15	T	C	0.489	-0.012	0.002	4.50E-09
**rs13009008**	2	A	G	0.328	0.012	0.002	4.60E-09
**rs17553262**	10	A	C	0.885	-0.018	0.003	5.30E-09
**rs7297175**	12	T	C	0.431	-0.012	0.002	6.60E-09
**rs245774**	5	A	G	0.272	-0.013	0.002	7.40E-09
**rs6962772**	7	A	G	0.846	0.016	0.003	7.80E-09
**rs12481282**	20	G	C	0.722	-0.013	0.002	7.80E-09
**rs35343344**	19	C	A	0.733	0.013	0.002	8.80E-09
**rs6562474**	13	C	G	0.651	0.012	0.002	1.00E-08
**rs775758**	3	A	T	0.433	0.012	0.002	1.10E-08
**rs2062882**	8	G	A	0.587	-0.012	0.002	1.10E-08
**rs7519626**	1	C	T	0.324	0.012	0.002	1.20E-08
**rs9435340**	1	T	A	0.344	0.012	0.002	1.20E-08
**rs34866095**	11	A	G	0.686	-0.012	0.002	1.20E-08
**rs348809**	20	A	G	0.348	-0.012	0.002	1.30E-08
**rs1050847**	16	C	T	0.426	0.011	0.002	1.40E-08
**rs73220544**	3	A	C	0.842	-0.016	0.003	1.50E-08
**rs4571506**	5	C	T	0.54	0.011	0.002	1.50E-08

**rs732083**	17	G	A	0.333	0.012	0.002	1.50E-08
**rs6741228**	2	T	C	0.433	0.011	0.002	1.60E-08
**rs4949465**	1	T	C	0.87	-0.017	0.003	1.70E-08
**rs62175972**	2	T	C	0.966	0.031	0.006	1.70E-08
**rs136233**	22	A	G	0.809	-0.014	0.003	1.80E-08
**rs12831617**	12	C	T	0.764	-0.013	0.002	1.90E-08
**rs11861214**	16	G	T	0.784	0.014	0.002	2.00E-08
**rs10918701**	1	G	A	0.372	0.012	0.002	2.10E-08
**rs10823968**	10	A	T	0.633	0.012	0.002	2.10E-08
**rs74086911**	12	G	A	0.925	0.021	0.004	2.10E-08
**rs4731925**	7	C	T	0.316	-0.012	0.002	2.60E-08
**rs28485305**	15	C	T	0.631	0.012	0.002	2.60E-08
**rs1193237**	1	G	C	0.439	-0.011	0.002	2.80E-08
**rs60952428**	16	T	C	0.909	0.019	0.003	3.00E-08
**rs9904288**	17	T	C	0.708	0.012	0.002	3.10E-08
**rs12967855**	18	A	G	0.331	0.012	0.002	3.10E-08
**rs2254710**	6	C	A	0.236	0.013	0.002	3.50E-08
**rs72674867**	8	A	T	0.765	0.013	0.002	3.80E-08
**rs1931263**	1	G	T	0.51	-0.011	0.002	4.00E-08
**rs57611503**	16	G	A	0.485	0.011	0.002	4.00E-08
**rs61796681**	4	A	T	0.912	-0.019	0.004	4.20E-08
**rs6957896**	7	C	T	0.503	-0.011	0.002	4.50E-08
**rs2080870**	5	A	T	0.258	0.012	0.002	4.90E-08

Full acknowledgement of the Melanoma GWAS meta-analysis

NCI

This study was supported by the Intramural Research Program of the Division of Cancer Epidemiology and Genetics, National Cancer Institute (NCI), National Institutes of Health (NIH) and Department of Health and Human Services (DHHS).

AOCS/OCAC/SEARCH

AOCS/OCAC/SEARCH is accessible via European Genome–Phenome Archive. We acknowledge their support and data, and the contribution of the study nurses, research assistants and all clinical and scientific collaborators in generation of these data. We also acknowledge their funding sources: OCAC (NIH grant no. U19CA148112), SEARCH team (Cancer Research UK grant no.C490/A16561), AOCS (US Army Medical Research and Material Command under grant no. DAMD17-01-1-0729, The Cancer Council Victoria, Queensland Cancer Fund, The Cancer Council New South Wales, The Cancer Council South Australia, The Cancer Foundation of Western Australia, The Cancer Council Tasmania and the National Health and Medical Research Council of Australia (NHMRC) (grant nos. ID400413 and ID400281, as well as support from S. Boldeman, the Agar family, Ovarian Cancer Action (UK), Ovarian Cancer Australia and the Peter MacCallum Foundation).

MelaNostrum Consortium

We thank the participants of the MelaNostrum Consortium from Italy (Genoa, L'Aquila, Rome, Padua, Milan, Florence and Bergamo), Spain (Valencia and Barcelona), Greece (Athens) and Cyprus (Nicosia) who provided data and biospecimens for this study. The Consortium is partially supported by the Intramural Research Program of the Division of Cancer Epidemiology and Genetics, NCI, NIH, DHHS. Funding for the University of Genoa and Genetics of Rare Cancers, Ospedale Policlinico San Martino came from Italian Ministry of Health 5 × 1000 per la Ricerca Corrente to Ospedale Policlinico San Martino and AIRC IG 15460. The research at the Melanoma Unit in Barcelona was supported by the Spanish Fondo de Investigaciones Sanitarias grant nos. PI15/00716 and PI15/00956 cofinanced by FEDER 'Una manera de hacer Europa'; CIBER de Enfermedades Raras of the Instituto de Salud Carlos III, Spain, cofinanced by European Development Regional Fund 'A way to achieve Europe' ERDF; AGAUR 2014_SGR_603 of the Catalan Government, Spain; European Commission, contract no. LSHC-CT-2006-018702 (GenoMEL) and by the European Commission under the 7th Framework Programme, Diagnostics; 'Fundació La Marató de TV3' grant no. 201331-30, Catalonia, Spain; 'Fundación Científica de la Asociación Española Contra el Cáncer' grant no. GCB15152978SOEN, Spain, and CERCA Programme/Generalitat de Catalunya. Melanoma research at the Department of Dermatology, University of L'Aquila, Italy was supported by the Italian Ministry of the University and Scientific Research (PRIN-2012 grant no. 2012JJX494).

Q-MEGA/QTWIN:

The Q-MEGA/QTWIN study was supported by the Melanoma Research Alliance, the NIH NCI (grant nos. CA88363, CA83115, CA122838, CA87969, CA055075, CA100264, CA133996 and CA49449), the NHMRC (grant nos. 200071, 241944, 339462, 380385, 389927, 389875, 389891, 389892,389938, 443036, 442915, 442981,

496610, 496675, 496739, 552485, 552498 and APP1049894), the Cancer Councils New South Wales, Victoria and Queensland, the Cancer Institute New South Wales, the Cooperative Research Centre for Discovery of Genes for Common Human Diseases, Cerylid Biosciences (Melbourne), the Australian Cancer Research Foundation, The Wellcome Trust (grant no. WT084766/Z/08/Z) and donations from N. and S. Hawkins. S. MacGregor acknowledges fellowship support from the Australian National Health and Medical Research Council and from the Australian Research Council."

Endometriosis
Contributors to the Endometriosis collection: Anjali K. Henders, S.H. Kennedy, S. Macgregor, N.G. Martin, S. Missmer, G.W. Montgomery, D.R. Nyholt, J.N. Painter, S.A. Treloar, L. Wallace, K.T. Zondervan. Acknowledgements: We acknowledge all the participants in the QIMR and endometriosis studies. We thank Anjali Henders, Leanne Wallace, and Lisa Bowdler for project management, sample processing and database development. We thank Endometriosis Associations for supporting study recruitment and S. Nicolaides and the Queensland Medical Laboratory for assistance with blood collection including pro bono collection and delivery of blood samples. Funding: This work was supported by the Cooperative Research Centre (CRC) for Discovery of Genes for Common Human Diseases, Cerylid Biosciences (Melbourne), The Wellcome Trust and donations from Neville and Shirley Hawkins. Endometriosis sample genotyping was funded by a grant from the Wellcome Trust (WT084766/Z/08/Z) and NHMRC (496610, GNT1049472, GNT1050208). G.W.M. is supported by the NHMRC Fellowships scheme. D.R.N. was supported by the NHMRC Fellowship (613674) and Australian Research Council (ARC) Future Fellowship (FT0991022) schemes.

EPIGENE

We thank melanoma patients, Sullivan and Nicolaides Pathology, Queensland Medical Laboratories, and IQ Pathology for their involvement and support. This cohort was supported by the National Health and Medical Research Council of Australia (APP442960).

QSkin

The QSkin Study could not have occurred without the valuable contribution of the Queenslanders who took part, and for that we thank them. The authors gratefully acknowledge the valuable contributions of all staff, students and colleagues who have been associated with the project since its inception. We would like to recognise Australian National Health and Medical Research Council funding (project grant APP106306, programme grant 552429)

Princess Alexandra Hospital (PAH) samples
We acknowledge the support and assistance of study participants, and Adele Greene (securing funding), Mark Smithers (cohort establishment), as well as Casey Rowe and Maryrose Malt for consenting and collection of patient samples. Funding for the PAH collection was via the University of Queensland Diamantina Institute, the Meehan Foundation, NHMRC CDF (1125290), and Cancer Council Queensland (1125237).

AMFS

This work was supported by the National Health and Medical Research Council of Australia (NHMRC) project grants 566946, 107359, 211172, and program grant number 402761 to GJM and RFK. Work was also funded by the Cancer Councils of Victoria, Queensland, and New South Wales (project grants 77/00, 06/10, 371) and by US NIH RO1 grant CA-83115-01A2 and 2R01CA083115-11A1. Anne E. Cust is supported by fellowships from the Cancer Institute NSW and the NHMRC. We gratefully thank the support and involvement of all participants, research coordinators, interviewers, examiners and data management staff.

Study of Digestive Health

Controls for use with the Q-MEGA_omni dataset were derived from the Study of Digestive Health group (SDH) which was funded by NCI grant 5 RO1 CA 001833-02. Its contents are solely the responsibility of the authors and do not necessarily represent the official views of the National Cancer Institute. We gratefully acknowledge the cooperation of the following institutions: Sullivan and Nicolaides Pathology (Brisbane); Queensland Medical Laboratory (Brisbane); Queensland Health Pathology Services (Brisbane); Institute of Medical and Veterinary Science (Adelaide); SouthPath (Adelaide). We also acknowledge the contribution of the study nurses and research assistants and would like to thank all of the people who participated in the study. DCW was supported by an NHMRC Research Fellowship (APP1058522).

Inflammatory Bowel Disease (IBD)

We gratefully acknowledge the aid in identifying study participants by Sullivan and Nicolaides Pathology, Queensland Medical Laboratories and the Queensland Health Pathology Service. The IBD study would like to recognise and thank Peter Schultz, Lauren Aoude, Loralie Parsonson, Stephen Walsh, Mitchell Stark, John Cardinal and Herlina Handoko for technical support.

This work was funded by US NCI grant CA 001833-03. Its contents are solely the responsibility of the authors and do not necessarily represent the official views of the National Cancer Institute. PMW and DCW are Senior Research Fellows of the National Health and Medical Research Council (NHMRC) of Australia. NP was supported by a NHMRC PhD scholarship. The funding bodies played no role in the design or conduct of the study, the collection, management, analysis, or interpretation of the data, or the preparation, review or approval of the manuscript.

Essen-Heidelberg

The study was supported by a grant from Deutsche Forshungsgemeinschaft (GZ: SCHA 422/11-1).

Western Australian Melanoma Health Study (WAMHS)

The WAMHS, and the salaries of its staff and PhD students, was funded by the Scott Kirkbride Melanoma Research Centre. The Cancer Council Western Australia is also acknowledged for current salary support for Sarah Ward (Capacity Building and Collaboration grant). We acknowledge the donation of time and samples from study participants, the WAMHS study team, and the WAMHS Management Committee. This work could not have

occurred without the assistance and support of the Western Australian (WA) DNA Bank, and the Ark at The University of WA, and the WA Cancer Registry, for which we are grateful.

Australian & New Zealand Registry of Advanced Glaucoma (ANZRAG)
Participant collection was funded by the Royal Australian and New Zealand College of Ophthalmology Eye Foundation. Genotyping was supported by NHMRC grants 535074 and 1023911. ANZRAG also acknowledges funding from the BrightFocus Foundation and a Ramaciotti Establishment Grant. The authors acknowledge the support of Ms. Bronwyn Usher-Ridge in patient recruitment and data collection, and Dr Patrick Danoy and Dr Johanna Hadler for genotyping.

Generations Scotland
Generation Scotland received core support from the Chief Scientist Office of the Scottish Government Health Directorates [CZD/16/6] and the Scottish Funding Council [HR03006]. Genotyping of the GS:SFHS samples was carried out by the Genetics Core Laboratory at the Wellcome Trust Clinical Research Facility, Edinburgh, Scotland and was funded by the Medical Research Council UK and the Wellcome Trust (Wellcome Trust Strategic Award "STratifying Resilience and Depression Longitudinally" (STRADL) Reference 104036/Z/14/Z)."

Michigan
The authors acknowledge the University of Michigan Precision Health Initiative and Medical School Central Biorepository for providing biospecimen storage, management, processing and distribution services and the Center for Statistical Genetics in the Department of Biostatistics at the School of Public Health for genotype data curation, imputation, and management in support of this research.

Brisbane Nevus Morphology Study
This work was funded by project grant numbers NHMRC 1004999, 1062935 and the Centre of Research Excellence for the Study of Naevi 1099021. This research was carried out at the Translational Research Institute, Woolloongabba, QLD 4102, Australia.

The Translational Research Institute is supported by a grant from the Australian Government

MIA
This work was supported by research funding from Melanoma Institute Australia, the National Health and Medical Research Council of Australia (NHMRC) through program grants to GJM, RAS & GVL and from Cancer Institute New South Wales and infrastructure grants from Macquarie University and the Australian Cancer Research Foundation. R.A.S. and G.V.L. are supported by NHMRC Fellowships, and G.V.L. is supported by the University of Sydney Medical Foundation. RS is supported by the Melanoma Institute Australia (MIA), the New South Wales Department of Health, NSW Health Pathology, the National Health and Medical Research Council of Australia (NHMRC) and Cancer Institute NSW and reports receiving fees for professional services from Merck Sharp &

Dohme, GlaxoSmithKline Australia, Bristol-Myers Squibb, Dermpedia, Novartis Pharmaceuticals Australia Pty Ltd, Myriad, NeraCare GmbH and Amgen.

Nevus GWAS meta-analysis dataset

For full funding support and acknowledgements see [10]. These are briefly summarised here. All studies gratefully acknowledge the support of all research participants and twins, research nurses, research assistants and support staff, without whom this work would not be possible. In addition to specific named thanks below, we also acknowledge the support of G. Clement, Bernet Keto, Pirro Hysi and Emad Qweitin.

Acknowledgements for GWAS contributing to the nevus meta-analysis are as follows:

ALSPAC: The UK Medical Research Council, Wellcome Trust (Grant ref: 102215/2/13/2), the University of Bristol, Sample Logistics and Genotyping Facilities at the Wellcome Trust Sanger Institute and LabCorp (Laboratory Corporation of America). 23andMe, ARC Future Fellowship (FT130101709), and a Medical Research Council program grant (MC_UU_12013/4).

Harvard: The NHS and HPFS cohorts, US State Cancer Registries (for the full list of states see [10]), the Odyssey cluster (FAS Division of Science, Research Computing Group at Harvard University.). Funding support by NIH R01 CA49449, P01 CA87969, UM1

CA186107, and UM1 CA167552.

Leeds: Funding from Cancer Research UK (project grant C8216/A6129 and programme award C588/A4994) and by the NIH (R01 CA83115). The UK National Cancer Research supported participant recruitment. We specifically acknowledge the support of Patricia Mack, Kate Gamble, Paul King, and Dr Amy Downing.

QIMR: We specifically thank Dixie Statham, Ann Eldridge, Marlene Grace, Kerrie McAloney, Natalie Garden, Reshika Chand, Lisa Bowdler, Leanne Wallace, David Smyth, Harry

Beeby, and Daniel Park. **The Brisbane Twin Nevus Study** was supported by Australian National Health and Medical Research Council (NHMRC) grants (241944, 339462, 389927, 389875, 389891, 389892, 389938, 442915, 442981, 496739, 552485, 552498, 1031119), Adult twin data collection by NIH grant (AA011998_5978), over-50 twins by a donation from Mr. George Landers of Chania, Crete. Genotyping was funded by the NHMRC (552498 and 1049894). We also acknowledge the U.S. National Institutes of Health's support through grants AA07535, AA10248, AA13320, AA13321, AA13326, AA14041, and MH66206. This work was also funded by the FP-5 GenomEUtwin Project (QLG2-CT-2002-01254). Genotyping for individuals using the Illumina 370K was funded by an access award to Dr. Richard Todd and performed at the Center for Inherited Disease Research, Baltimore. Nick Hayward, David C. Whiteman, David. L. Duffy, Grant W. Montgomery were supported by the NHMRC Fellowships scheme during the collection of these samples.

Raine: We acknowledge the support for cohort coordination and data collection from theRaine Study and Lions Eye Institute. The Raine Study is supported and funded by The University of Western Australia (UWA), The Telethon Institute for Child Health Research, Raine Medical Research Foundation, UWA Faculty of Medicine, Dentistry and Health Sciences, Women's and Infant's Research Foundation and Curtin University. Genotyping was funded by the NHMRC (1021105). Support for the REHS was provided by LEI, the Australian Foundation for the Prevention of Blindness and the Ophthalmic Research Institute of Australia.

The Rotterdam Study: We specifically acknowledge the assistance and support of Ada Hooghart, Corina Brussee, Riet Bernaerts-Biskop, Patricia van Hilten, Pascal Arp, Jeanette Vergeer, Maarten Kooijman and Lennart Karssen. The Rotterdam study was supported by the Netherlands Organisation of Scientific Research (NWO); Erasmus Medical Center and Erasmus University, Rotterdam, The Netherlands; Netherlands Organization for Health Research and Development (ZonMw); UitZicht; the Research Institute for Diseases in the Elderly; the Ministry of Education, Culture and Science; the Ministry for Health, Welfare and Sports; the European Commission (DG XII); the Municipality of Rotterdam; the Netherlands Genomics Initiative/NWO; Center for Medical Systems Biology of NGI; Stichting Lijf en Leven; Stichting Oogfonds Nederland; Landelijke Stichting voor Blinden en Slechtzienden; Algemene Nederlandse Vereniging ter Voorkoming van Blindheid; Medical Workshop; Heidelberg Engineering; Topcon Europe BV. Research Institute for Diseases in the Elderly (014-93-015; RIDE2), the Netherlands Genomics Initiative (NGI)/Netherlands Organisation for Scientific Research (NWO) project nr. 050-060-810.

F.L. was supported by the Erasmus University Rotterdam (EUR) fellowship and the Chinese recruiting program "The 1000 Talents Plan" for young scholars. GWAS genotyping was funded by the Netherlands Organisation of Scientific Research NWO Investments (nr. 175.010.2005.011, 911-03-012).

Twins eye study in Tasmania: This work was supported by an NHMRC Enabling Grant (2004-2009, 350415, 2005-2007); Clifford Craig Medical Research Trust; Ophthalmic Research Institute of Australia; American Health Assistance Foundation; Peggy and Leslie Cranbourne Foundation; Foundation for Children; Jack Brockhoff Foundation, and the National Eye Institute of the National Institute of Health grants RO1EY01824601 from 2007 to 2010. D. Mackey was supported by a Pfizer Australia Senior Research Fellowship and S. MacGregor was a recipient of an Australian NHMRC Career Development Award. Genotyping was funded by an NHMRC Medical Genomics Grant; US NIH/National Eye Institute (1RO1EY018246), Australian sample imputation analyses were carried out on the Genetic Cluster Computer, which is financially supported by the Netherlands Scientific Organization (NWO48005003).

TwinsUK: TwinsUK specifically thanks U. Perks, and acknowledges the funding support from the Wellcome Trust, Medical Research Council, European Union, the National Institute for Health Research (NIHR)-funded BioResource, Clinical Research Facility and Biomedical Research Centre based at Guy's and St Thomas' NHS Foundation Trust in partnership with King's College London, and . T.D.S. is an NIHR senior investigator and D.G is an MRC clinical research fellow. Genotyping was supported by the National Eye Institute via an NIH/CIDR genotyping project (PI: Terri Young), the Genotyping Facilities at the Wellcome Trust Sanger Institute.

ENGAGE: We thank the members of the Engage consortium for providing the telomere GWAS data reported in [34]; please see this publication for acknowledgements relevant to this dataset.

MDACC: We thank the individuals who volunteered to participate in this project. This work was supported by the National Cancer Institute of the National Institutes of Health through SPORE grant P50 CA093459 and Cancer Center Support Grant P30 CA016672 (Clinical Trials Support Resource), as well as by philanthropic contributions to The University of Texas MD Anderson Cancer Center Moon Shots Program, The University of Texas MD Anderson Cancer Center Various Donors Melanoma and Skin Cancers Priority Program Fund, the Miriam and Jim Mulva Research Fund, the McCarthy Skin Cancer Research Fund, and the Marit Peterson Fund for Melanoma Research.

Genoa: The Genoa study was supported by The Italian Ministry of Health Grant RF-2016-02362288 and 5x1000 per la ricerca corrente. The authors acknowledge Dr. William Bruno for his continuous work on melanoma families.

Barcelona: The research at the Melanoma Unit in Barcelona is partially funded by Spanish Fondo de Investigaciones Sanitarias grants PI15/00716, PI15/00956, PI18/00419 and PI18/01077 ; CIBER de Enfermedades Raras of the Instituto de Salud Carlos III, Spain, co-financed by European Development Regional Fund "A way to achieve Europe" ERDF; AGAUR 2017_SGR_1134 of the Catalan Government, Spain; European Commission under the 6th Framework Programme, Contract No. LSHC-CT-2006-018702 (GenoMEL) and by the European Commission under the 7th Framework Programme, Diagnoptics; The National Cancer Institute (NCI) of the US National Institute of Health (NIH) (CA83115); a grant from "Fundació La Marató de TV3" 201331-30, Catalonia, Spain; a grant from "Fundación Científica de la Asociación Española Contra el Cáncer" GCB15152978SOEN, Spain, and CERCA Programme / Generalitat de Catalunya. Part of the work was carried out at the Esther Koplowitz Center, Barcelona.

ICR: We thank Breast Cancer Now and the Institute of Cancer Research for funding and acknowledge National Health Service funding to the Royal Marsden NHS Foundation Trust and Institute of Cancer Research NIHR Biomedical Research Centre.

MELARISK, France: This work was supported by grants from Institut National du Cancer (INCa-PL016 and INCa_5982) to FD, Ligue Nationale Contre Le Cancer (PRE 09/FD) to FD, Programme Hospitalier de Recherche Clinique (AOM-07-195) to MFA and FD, Ministère de l'Enseignement Supérieur et de la Recherche and Institut National du Cancer (INCa) to GML. MB was supported by fellowships from Ligue Nationale Contre Le Cancer and Fondation pour la Recherche Médicale (FDT20130928343). The authors thank the French Family study

group for contributing data to the MELARISK study3,5,64, the Supplementation in Vitamins and Mineral Antioxidants (SU.VI.MAX) study group for giving access to data of the Su.VI.MAX study65 and the Epidemiological Study on the Genetics and Environment of Asthma (EGEA) cooperative group for giving access to data of the EGEA study (https://egeanet.vjf.inserm.fr). We acknowledge that the biological specimens of the French MELARISK study were obtained from the Institut Gustave Roussy and Fondation Jean Dausset–CEPH Biobanks.
